# Substituent effects in hydrogen bonding: DFT and QTAIM studies on acids and carboxylates complexes with formamide

**DOI:** 10.1007/s00894-014-2356-8

**Published:** 2014-07-15

**Authors:** Borys Ośmiałowski

**Affiliations:** Faculty of Chemical Technology and Engineering, University of Technology and Life Sciences, Seminaryjna 3, PL-85-326 Bydgoszcz, Poland

**Keywords:** Cooperativity, Hydrogen bonding, QTAIM theory, Substituent effect, Weak interactions

## Abstract

Four series of hydrogen bonded complexes of formamide and substituted benzoic acids and benzoates were studied in the light of substituent effect on intermolecular interactions. The analysis based on energy of interaction, geometry, QTAIM-derived properties of hydrogen bond critical point and energy of hydrogen bonds were made and discussed. The opposite effect of the substituent on hydrogen bond donor and acceptor in acid series was found and analyzed. The isodesmic reactions were used to further study the interaction preferences.

**Electronic supplementary material:**

The online version of this article (doi:10.1007/s00894-014-2356-8) contains supplementary material, which is available to authorized users.

**Figure:**
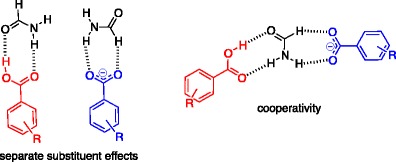
ᅟ

## Introduction

**Fig. 1:**
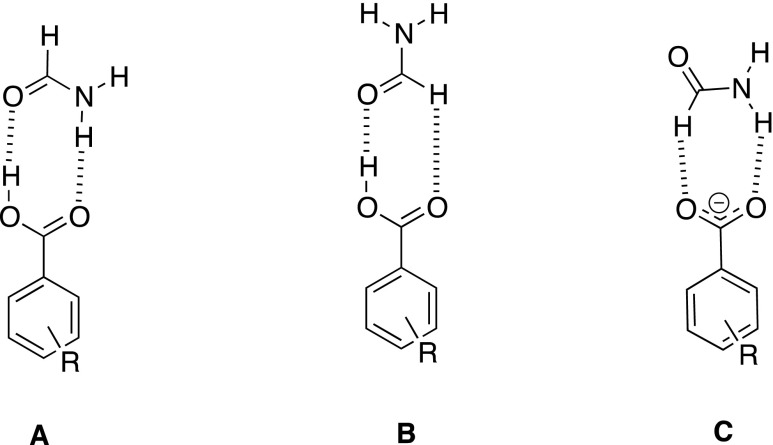
Three forms of studied formamide complexes

Nowadays hydrogen bonding is used as a force in non-covalent synthesis. This synthesis [1] is focused mostly on crystal engineering [2–4], a non-covalent polymerization [5–7] but hydrogen bonding may be used also in chemical sensing [8–14]. The ability to tune the properties of molecules by changing the substituent comes from the basic research by Hammett [15]. However, in supramolecular chemistry there are several possibilities to tune the non-covalent interactions [16, 17]. These are the electronic substituent effect [18–20, 17], steric effect [21–24], and intermolecular electronic repulsion [25] to mention a few. Although very good reviews on tuning the molecular properties and thus influence on their interactions has been published there are still not so many extensive works in the topic of pure substituent effect in simple associates. This is especially true for studies taking into account more than five various substituents. Recently it was shown that the association of substituted benzoates with heterocyclic urea derivatives preceded by breaking the intramolecular hydrogen bond is driven by the character of the substituent [18]. In compounds that are able to form a intramolecular hydrogen bond such a conformation is preferred. There are many examples of compounds that behave this way as: heterocyclic ureas [26, 27], enamines or enolimines [28–30] and others including biomolecules [31]. On the other hand the compounds that are not able to form such stabilizing interaction can form various dimers, trimers etc. or their rotamers may be stabilized as various associates. The rotamerism in such molecules may be probed by the use of appropriate hydrogen-bonding counterparts [21]. However in simple, model molecules such rotamerism is not possible or does not influence the association due to the symmetry of the part of the molecule. The rotation about C-N bond in formamide does not change its hydrogen-bonding pattern. Thus this simple model may be a structure of choice [32] to study the interactions of amides with acids and carboxylates taking into account the substituent effect. The carboxylates were used in several studies as anions that probe the non-covalent interactions [19, 33, 34]. The regular substituent-dependent changes in properties of carboxylic acids and thus benzoates are commonly known. The acidity and thus donation ability of the hydrogen bond donor (OH) in -COOH moiety in acids may be a good test for description of interaction preferences driven by the character of the substituent. On the other hand the basicity and thus the ability of being an acceptor for hydrogen bonds as in phenolates [35–38] of carboxylates [19, 33, 34] may be used in a similar way. In 4-substituted benzoates, however, both oxygen atoms are equal in the light of hydrogen bonding. In acids the -COOH group contains donor and acceptor of the hydrogen bond. Changing the electronic properties of the carboxylic acid influence the properties of C = O and OH groups in opposite ways. These features were taken into account during design of the series of calculations. The aim of this study is to: *a*) study the substituent effect on association of the model molecule (formamide), *b*) check if the weak hydrogen bond by CH group plays a crucial role in association, *c*) compared to the various arrangements of molecules within the complex in light of their interaction and *d*) study how the substituent effect influences association when two various species are associated with formamide at a time. In Fig. 1 three forms of associates are shown.

It is worth pointing out that forms **A**, **B**, and **C** differ by topology. In form **A** the eight-membered ring is stabilized by two hydrogen bonds, while in **B** the seven-membered ring is present. In form **C** the seven-membered ring is also stabilized by two hydrogen bonds but it differ from **B** by *a*) net charge and *b*) the composition of the quasi-ring with hydrogen bonds. In forms **A** and **B** both molecules consist of one hydrogen bond acceptor and one donor (assuming CH · · · O weak interaction as a hydrogen bond type of interaction), while in **C** both acceptors belong to benzoate and both hydrogen bond donors to the formamide.

## Computational methods

All calculations were performed with the use of Gaussian software [39]. The M05 functional suggested for non-covalent interactions [40, 41] was used together with the 6-311+G(2d,2p) basis set as in previous publications [18, 21, 22]. The use of the diffuse functions is crucial for describing the anionic specie, while polarization functions are used to properly describe the hydrogen bonding. Optimizations were performed with the use of PCM model of solvation [42] in chloroform since in previous publications such treatment was in agreement with experimental results [18, 21, 22]. For all optimized structures the frequency calculations were ran to check if the geometry is in energy minima (only positive frequencies were obtained). The quantum theory of atoms in molecules (QTAIM) derived data were calculated with the AIM2000 software [43]. The intermolecular interaction energies were calculated with the use of basis set superposition error correction (BSSE) using counterpoise procedure [44, 45] with default settings. These were single point calculations at the geometry optimized in the previous step. All energies are zero point energy (ZPE) corrected.

## Results and discussion

The optimized structures of complexes allowed analyzing the geometry, interaction energy and electronic parameters of complexes. Table 1 collects the crucial intermolecular distances for forms **A**-**C** and the substituent constants (σ) to show the tendency of changes.

**Table 1:** The lengths [Å] of hydrogen bonds in complexes **A**-**C**

		Form **A**		Form **B**		Form **C**	
R	σ [46]	OH · · · O	NH · · · O	OH · · · O	CH · · · O	CH · · · O	NH · · · O
NMe_2_	−0.83	1.739	1.919	1.732	2.454	2.662	1.798
NH_2_	−0.66	1.732	1.925	1.725	2.464	2.663	1.801
OMe	−0.27	1.719	1.935	1.714	2.462	2.663	1.810
Me	−0.17	1.715	1.941	1.710	2.485	2.666	1.807
H	0.00	1.708	1.946	1.704	2.491	2.668	1.811
F	0.06	1.703	1.949	1.699	2.496	2.669	1.814
Cl	0.23	1.700	1.953	1.695	2.501	2.674	1.817
CF_3_	0.54	1.690	1.962	1.685	2.515	2.682	1.821
NO_2_	0.78	1.679	1.971	1.674	2.525	2.696	1.830
*R*		0.998	0.998	0.998	0.970	0.899	0.983

*R* - correlation coefficient

The above data shows that the substituent influences intermolecular distances in a classical way—the interatomic distances are in line with the substituent constant, which in turn describes the systematic electronic effect of the substituent on properties of acid/base. The high correlation coefficients (*R*, last row) confirm that the model of linear changes of properties of molecules works also for intermolecular contacts. The smaller value of correlation coefficient for CH · · · O interaction (form **C**) suggest this contact is weak and acts as a support for the stronger NH · · · O one allowing interacting parts of molecules to occupy a common plane. The CH · · · O hydrogen bond length in form **B** correlates better with substituent constant than that in **C**. This may be caused by the increased strength of OH · · · O hydrogen bond with respect to the NH · · · O one (the OH · · · O is, in general [47], stronger than NH · · · O hydrogen bond—form **B** vs. **C**). Also the CH · · · O hydrogen bond distances suggest this interaction in **B** is stronger than in **C**. The intermolecular interaction energies (E_int_) corrected to ZPE and BSSE [44] are collected in Table 2. The same table contains the correlation coefficient (*R*) for E_int_ = *a* (σ) + *b* function and *a*, *b*, and σ (substituent constant) values.

**Table 2:** ZPE and BSSE corrected E_int_ [kJ mol^-1^] for the studied complexes

R	σ [46]	Form **A**	Form **B**	foRm **C**
NMe_2_	−0.83	−37.7	−28.8	−40.7
NH_2_	−0.66	−37.9	−29.2	−40.3
OMe	−0.27	−38.6	−30.2	−39.4
Me	−0.17	−38.7	−30.8	−39.3
H	0	−39.1	−31.5	−38.8
F	0.06	−39.5	−32.1	−38.4
Cl	0.23	−39.7	−32.4	−38.1
CF_3_	0.54	−40.3	−33.5	−37.6
NO_2_	0.78	−41.1	−34.7	−36.6
*R*		0.991	0.994	0.994
*a*		−2.12	−3.69	2.44
*b*		−39.25	−31.60	−38.72

*R* - correlation coefficient

It is interesting that the response of the E_int_ to the substituent is stronger in **B** form with respect to the **A** form by the factor of 1.74 (−3.69/−2.12 = 1.74). This is expressed by steeper linear function for **B** than that for **A** (compare the ‘*a*’ values in Table 2). It is worth pointing out that ‘*a*’ (Table 2) have opposite sign for acid series than that for anion series. This is in agreement with the acid/base properties of these species, i.e., the electron-accepting substituent increase the acidity of an acid and lowers the basicity of the conjugated base. In order to get a deeper insight into the properties of the studied complexes the QTAIM [48] analysis was performed. Tables 3, 4 and 5 collect the data of Laplacian of the electron density (∇^2^ *ρ*), electron density (*ρ*) at the hydrogen bond critical point (H-BCP) and the energy of the hydrogen bond (E_HB_) calculated by the Espinosa approach [49, 50]. The ∇^2^ *ρ* and *ρ* values are limited to three decimals for stronger interactions and four for weaker ones. Similarly, the values of E_HB_ are limited to one decimal for stronger interactions and two for weaker ones. This is to show numerically the tendency of changes upon change of the substituent. The last three rows collect correlation coefficients (*R*) for the following linear function property (∇^2^ *ρ*, *ρ* or E_HB_) = *a* (σ) + *b* and *a* (slope) and *b* (intercept) values.

**Table 3:** The ∇^2^ *ρ*, *ρ* and E_HB_ [kJ mol^-1^] of hydrogen bonds in complexes **A**

	OH · · · O interaction			NH · · · O interaction		
	∇^2^ *ρ*	*ρ*	E_HB_	∇^2^ *ρ*	*ρ*	E_HB_
NMe_2_	0.113	0.039	−42.8	0.086	0.026	−25.6
NH_2_	0.114	0.040	−43.7	0.085	0.026	−25.2
OMe	0.117	0.041	−45.3	0.083	0.025	−24.4
Me	0.118	0.041	−45.9	0.082	0.025	−24.0
H	0.119	0.042	−46.7	0.082	0.025	−23.7
F	0.120	0.042	−47.5	0.081	0.024	−23.5
Cl	0.121	0.043	−48.0	0.081	0.024	−23.2
CF_3_	0.123	0.044	−49.4	0.079	0.024	−22.6
NO_2_	0.125	0.045	−51.0	0.078	0.023	−22.1
*R*	0.998	0.998	0.998	0.998	0.998	0.998
*a*	0.0075	0.0035	−5.015	−0.0052	−0.0018	2.175
*b*	0.1190	0.0419	−46.87	0.0817	0.0247	−23.73

**Table 4:** The ∇^2^ρ, *ρ* and E_HB_ [kJ mol^-1^] of hydrogen bonds in complexes **B**

	OH · · · O interaction			CH · · · O interaction		
	∇^2^ *ρ*	*ρ*	E_HB_	∇^2^ *ρ*	*ρ*	E_HB_
NMe_2_	0.116	0.040	−43.9	0.0335	0.0101	−8.27
NH_2_	0.117	0.040	−44.8	0.0329	0.0099	−8.12
OMe	0.119	0.042	−46.3	0.0330	0.0100	−8.15
Me	0.120	0.042	−46.9	0.0316	0.0095	−7.77
H	0.122	0.043	−47.7	0.0312	0.0094	−7.67
F	0.123	0.043	−48.5	0.0309	0.0093	−7.60
Cl	0.123	0.043	−49.1	0.0306	0.0092	−7.52
CF_3_	0.125	0.044	−50.5	0.0298	0.0090	−7.31
NO_2_	0.128	0.046	−52.2	0.0292	0.0088	−7.16
*R*	0.998	0.997	0.997	0.966	0.969	0.968
*a*	0.0072	0.0035	−5.043	−0.0028	−0.0009	0.715
*b*	0.1217	0.0427	−47.95	0.0313	0.0094	−7.70

**Table 5:** The ∇^2^ρ, ρ and E_HB_ [kJ mol^-1^] of hydrogen bonds in complexes **C**

	CH · · · O interaction			NH · · · O interaction		
	∇^2^ρ	ρ	E_HB_	∇^2^ρ	ρ	E_HB_
NMe_2_	0.0218	0.0075	−5.74	0.105	0.036	−37.1
NH_2_	0.0217	0.0074	−5.72	0.105	0.036	−36.8
OMe	0.0218	0.0074	−5.74	0.103	0.035	−35.8
Me	0.0216	0.0074	−5.68	0.104	0.035	−36.1
H	0.0215	0.0073	−5.65	0.103	0.035	−35.8
F	0.0214	0.0073	−5.63	0.102	0.035	−35.4
Cl	0.0212	0.0072	−5.57	0.102	0.035	−35.1
CF_3_	0.0209	0.0071	−5.48	0.101	0.034	−34.6
NO_2_	0.0204	0.0069	−5.30	0.100	0.033	−33.8
*R*	0.898	0.919	0.907	0.980	0.986	0.985
*a*	−0.0008	−0.0003	0.254	−0.0032	−0.0016	1.949
*b*	0.0213	0.0073	−5.60	*0*.1026	0.0349	−35.55

**Fig. 2:**
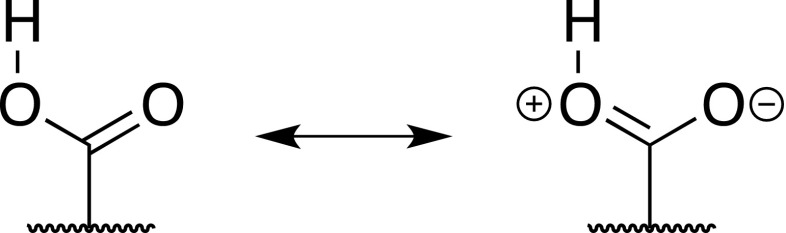
Mesomeric forms of -COOH group

First of all it is necessary to mention that in all complexes formed the values of the Laplacian of the electron density at H-BCP show the interaction is of hydrogen bond nature [51]. The QTAIM) theory says the Laplacian is negative for covalent bonds and positive for hydrogen bonds. It is commonly known that the -COOH moiety acts as a hydrogen bond donor (OH group) and acceptor (C = O group) at the same time. As the substituent became more electron-accepting it makes the hydrogen bond donation ability of OH higher and hydrogen bond accepting ability of C = O lower. The opposite signs of the ‘*a*’ values in E_HB_ columns show that (Tables 3 and 4), while the said slopes are positive for complexes **C** (Table 5). Due to the different electronegativity of nitrogen and carbon atoms the difference between NH · · · O and CH · · · O is obvious making the latter interaction weaker. The OH · · · O interaction is about 1.0 kJ mol^-1^ stronger in **B** form than that in **A** form. This suggests that the weak CH · · · O interaction supports five to seven times stronger OH · · · O one, while the NH · · · O in **A** complexes participates much more in overall stabilization. The relative E_HB_ energy in complexes **A** (E_HB_^(OH···O)^/E_HB_^(NH···O)^) and **B** (E_HB_^(OH···O)^/E_HB_^(CH···O)^) is the lowest for NMe_2_ and the highest for NO_2_ groups, respectively. Moreover, the said ratios defined as x_1_ = E_HB_(OH · · · O)/E_HB_(NH · · · O) in series **A** and *x*_2_ = E_HB_(OH · · · O)/E_HB_(CH · · · O) in series **B** are dependent from the substituent constants as: x_1_ = 0.39σ + 1.98 (*R* = 0.997) and *x*_2_ = 1.23σ + 6.25 (*R* = 0.989). The higher slope in the second equation confirms that CH · · · O interaction is weak while the slope in the first equation suggests the NH · · · O is much stronger than the CH · · · O one in series **B** (for equal contribution of interactions the slope should be equal to zero and the intercept equal to one). The E_HB_ of OH · · · O in **A** and **B** series are very similar. The above observations can also be seen in the geometry of the complexes (Table 1). The electron densities at H-BCPs in studied series are in line with the general trends in hydrogen bonding abilities of respective groups. The QTAIM data and above analysis gives the basis to the conclusion that two mesomeric forms of the -COOH moiety (Fig. 2) have their contributions dependent on the substituent and the hydrogen-bonding counterpart. The electron density on both oxygen atoms is, of course, dependent on substituent (SI, Chart S1). Increased E_HB_ for OH · · · X and decreased for H · · · O = C hydrogen bonds suggests the charge is transmitted within the -COOH group.

It is also worth mentioning that in associates the charge at the formamide proton forming hydrogen bond slightly increases (SI, Chart S2) with the increasing electron accepting properties of the substituent. This may be explained by the intra-COOH group conjugation.

**Fig. 3:**
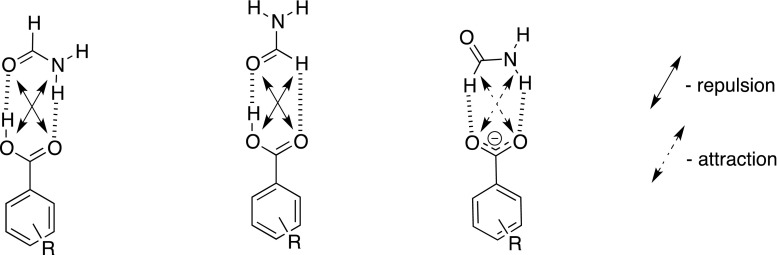
The attractive and repulsive secondary interactions in studied complexes

In the third series (complexes **C**) the E_int_ data shows that more electron-withdrawing substituent lowers the basicity of the carboxylate and thus weakens association. As in previous series the ∇^2^ *ρ* is positive exhibiting the hydrogen bonding nature of interaction. Similarly as in series **B** its value and the value of *ρ* are almost an order of magnitude lower for weak interaction with CH proton than that with much stronger interacting OH and NH ones. Also, as in **B** series, the values of *R* are lower for interaction with CH than that with NH. The slopes for all QTAIM-derived data in series **C** fitted to the linear function of the substituent constant are negative, which is in agreement with the general influence of the substituent on basicity. The change in E_HB_ of NH · · · O in series **A** and **C** is similar (compare the slopes in Tables 3 and 5). This means that these groups behave similarly independently if the interacting ‘X’ atom in NH · · · X hydrogen bond is neutral or has an anionic character as in series **C** however an increase of interaction is noticed for anions. The geometric and energetic parameters in series **C** correlate with substituent constants. Also, as before, the correlation is higher for a strong interaction (NH · · · O) than for a weak one (CH · · · O). It was recently shown [18] that the interaction with substituted benzoates is driven by the character of the substituent when associated with urea by two, close in energy NH · · · O hydrogen bonds. Here the NH · · · O hydrogen bond is much stronger (more than six times) than the CH · · · O interaction. The last interaction still follows the trend of the value of substituent constant. It is worth noting that the E_int_ for complexes in **C** series is higher than any E_int_ in **B**, while the former have comparable E_int_ to series **A** but with reversed order of changes in the light of substituent effect. On the other hand the sum of energies of hydrogen bonds in **C** is smaller than that in **B** (compare also the E_int_ values). This is caused by described earlier differences in topology of the complexes and hydrogen bonding patterns. In series **C** all intermolecular interactions are attractive while in **A** and **B** there are also repulsions (Fig. 3 shows secondary interactions). The concept of the secondary interactions [52] as forces that fulfill the palate of intermolecular interactions has been generally accepted by chemists. It says that in hydrogen-bonded complexes weak secondary interactions act diagonally within a cyclic system stabilized by hydrogen bonds. Thus, like groups repel while opposite in character attract each other. Solid and dashed arrows as in Fig. 3 usually depict these interactions.

**Fig. 4:**
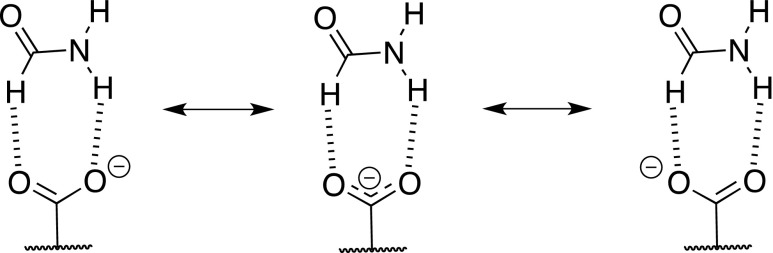
The mesomerism in carboxylate in hydrogen-bonded complex

The data for the C-O bond lengths in benzoate suggest these are not equivalent as they are in the case of isolated molecules. The NH · · · O hydrogen bonded oxygen atom forms a 0.012 Å longer bond to carbon atom than that of the oxygen entangled by CH · · · O interaction. Although the difference is very small it is reasonable to conclude that the CH · · · O interacting oxygen forms a little bit more double in character bond to carboxylic carbon atom than NH · · · O hydrogen bonded oxygen. This may also be interpreted as the subtle balance between the mesomeric forms present in the complex of carboxylate (Fig. 4). The mentioned data show that the structure on the left hand-side is a little bit more important that the one on the right hand-side.

To further study the driving forces in complexation of formamide the reactions expressed by equations 1–3 were used. Table 6 collects the energetic data.

**Table 6:** Energies [kJ mol^-1^] (ΔE_1−3_) for reactions 1–3

R	ΔE_1_	ΔE_2_	ΔE_3_
NMe_2_	−3.0	−11.8	8.9
NH_2_	−2.4	−11.1	8.7
OMe	−0.8	−9.1	8.4
Me	−0.6	−8.4	7.9
H	0.2	−7.4	7.6
F	1.1	−6.3	7.4
Cl	1.5	−5.8	7.3
CF_3_	2.7	−4.1	6.8
NO_2_	4.5	−1.9	6.4
*R*	0.993	0.995	0.989

1 $$ \mathrm{Form}\mathbf{A}+\mathrm{anion}\to \mathrm{Form}\mathbf{C}+\mathrm{acid}+\Delta {\mathrm{E}}_1 $$

2 $$ \mathrm{Form}\mathbf{B}+\mathrm{anion}\to \mathrm{Form}\mathbf{C}+\mathrm{acid}+\Delta {\mathrm{E}}_2 $$

3 $$ \mathrm{Form}\mathbf{A}+\mathrm{anion}\to \mathrm{Form}\mathbf{C}+\mathrm{acid}+\Delta {\mathrm{E}}_3 $$

The negative values in Table 6 show that reactions are exothermic (ΔE_2_), mixed depending on substituent (ΔE_1_), while the ΔE_3_ values suggest change from form **A** to **B** is not preferred. It is worth pointing out that the E_int_(**B**) and E_int_(**C**) (Table 2) change in opposite ways referring to the substituent constant order. Also these is no surprise that form **B** converts into **C** readily exchanging two repulsive interactions by two attractive (Fig. 3) although one hydrogen bond (CH · · · O in **C**) is weak.

**Fig. 5:**
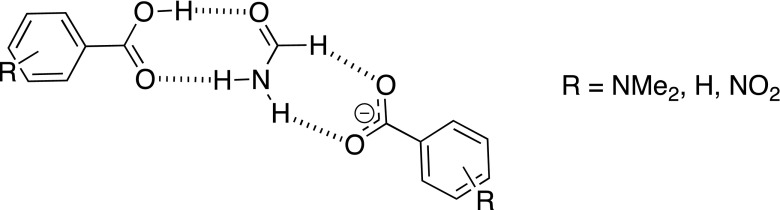
The association of formamide by the acid and benzoate (D associates)

Since the acid/benzoate equilibrium may be tuned by pH the simultaneous association by the acid and benzoate at a time may be considered. Thus, to have a better understanding of the substituent effect on association of acid and benzoate with formamide a series of calculations were employed. Figure 5 shows the associates that were investigated. Due to time consuming calculations only three substituents were used, i.e., extreme donor and acceptor and a neutral one.

Table 7 collects the E_HB_ for the complexes shown in Fig. 5. The values of ∇^2^ *ρ* and *ρ* are collected in supplementary material.

**Table 7:** The hydrogen bond energy (E_HB_ [kJ mol^-1^]) in complexes of formamide with acids and benzoates **D**

Acid side			Anion side		
E_HB_ [kJ mol^-1^]			E_HB_ [kJ mol^-1^]		
R	OH · · · O	NH · · · O	NH · · · O	CH · · · O	R
NO_2_	−63.6	−17.8	−36.2	−6.8	NO_2_
NO_2_	−65.2	−17.1	−38.5	−7.3	H
NO_2_	−66.0	−17.2	−39.8	−7.6	NMe_2_
H	−57.9	−17.8	−35.0	−6.7	NO_2_
H	−59.7	−17.0	−36.2	−7.9	H
H	−59.6	−17.1	−38.9	−7.2	NMe_2_
NMe_2_	−51.6	−19.4	−34.2	−5.1	NO_2_
NMe_2_	−53.6	−18.7	−35.8	−7.1	H
NMe_2_	−54.7	−17.5	−35.8	−8.0	NMe_2_

**Fig. 6:**
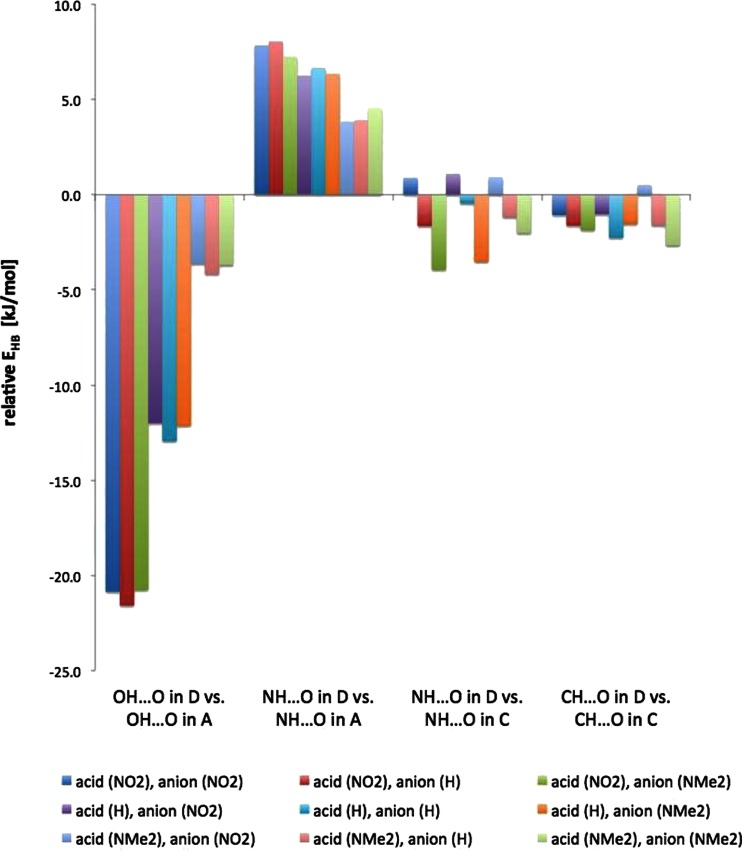
The changes in E_HB_ upon complexation of formamide with another counterpart

The data in Table 7 show some interesting features. The cooperativity effect [53] is noticed, i.e.: *a*) the OH · · · O interaction of acid became much stronger in **D** complexes than in complex **A**, *b*) consequently the NH · · · O interaction in **D** involving acid became weaker than in form **A**, *c*) except for NO_2_ substituted anion the NH · · · O with benzoate is stronger in **D** complexes than in form **C**, *d*) for the hydrogen bonding with acids and keeping the acid the same the extreme change of E_HB_ is observed for unsubstituted anion (E_HB_^(acid)^ in **D** vs. E_HB_^(acid)^ in **A**). These observations are depicted in Fig. 6. Negative values in Fig. 6 mean stronger interaction in **D** complex than in **A** or **C** forms. Here only the series **A** and **C** were compared to **D** because the topology of the **D** series is a superposition of topologies of **A** and **C** ones.

The said increase in energy of interaction has consequences on intermolecular distances. Table 8 collects the hydrogen-bond lengths in **D** associates.

**Table 8:** The hydrogen bond distances in **D** associates

Acid side			Anion side		
Hydrogen bond length [Å]			Hydrogen bond length [Å]		
R	OH · · · O	NH · · · O	NH · · · O	CH · · · O	R
NO_2_	1.611	2.058	1.806	2.566	NO_2_
NO_2_	1.604	2.073	1.786	2.531	H
NO_2_	1.600	2.072	1.775	2.517	NMe_2_
H	1.642	2.058	1.817	2.573	NO_2_
H	1.633	2.075	1.807	2.495	H
H	1.633	2.073	1.782	2.543	NMe_2_
NMe_2_	1.678	2.023	1.822	2.697	NO_2_
NMe_2_	1.668	2.039	1.811	2.551	H
NMe_2_	1.660	2.062	1.807	2.481	NMe_2_

**Fig. 7:**
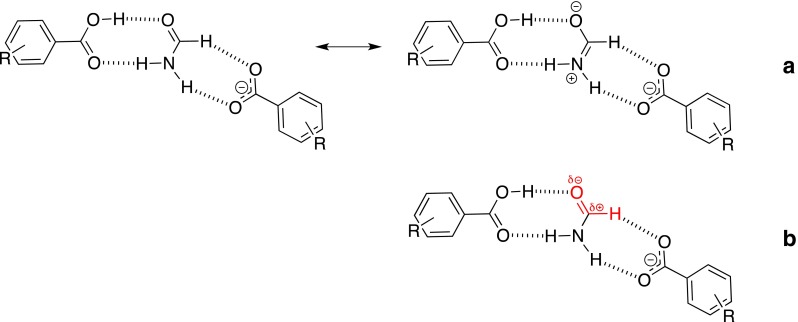
Two possible polarizations of the formamide molecule in D associates

Interestingly the energetic and geometrical data in **D** complexes suggest the polarization of the CH (Fig. 7*b*) bond rather than charge transfer from nitrogen to oxygen atom (mesomerism, Fig. 7*a*). This explains why the weak CH · · · O interaction with anions in **D** is stronger than that in **C** series.

**Fig. 8:**
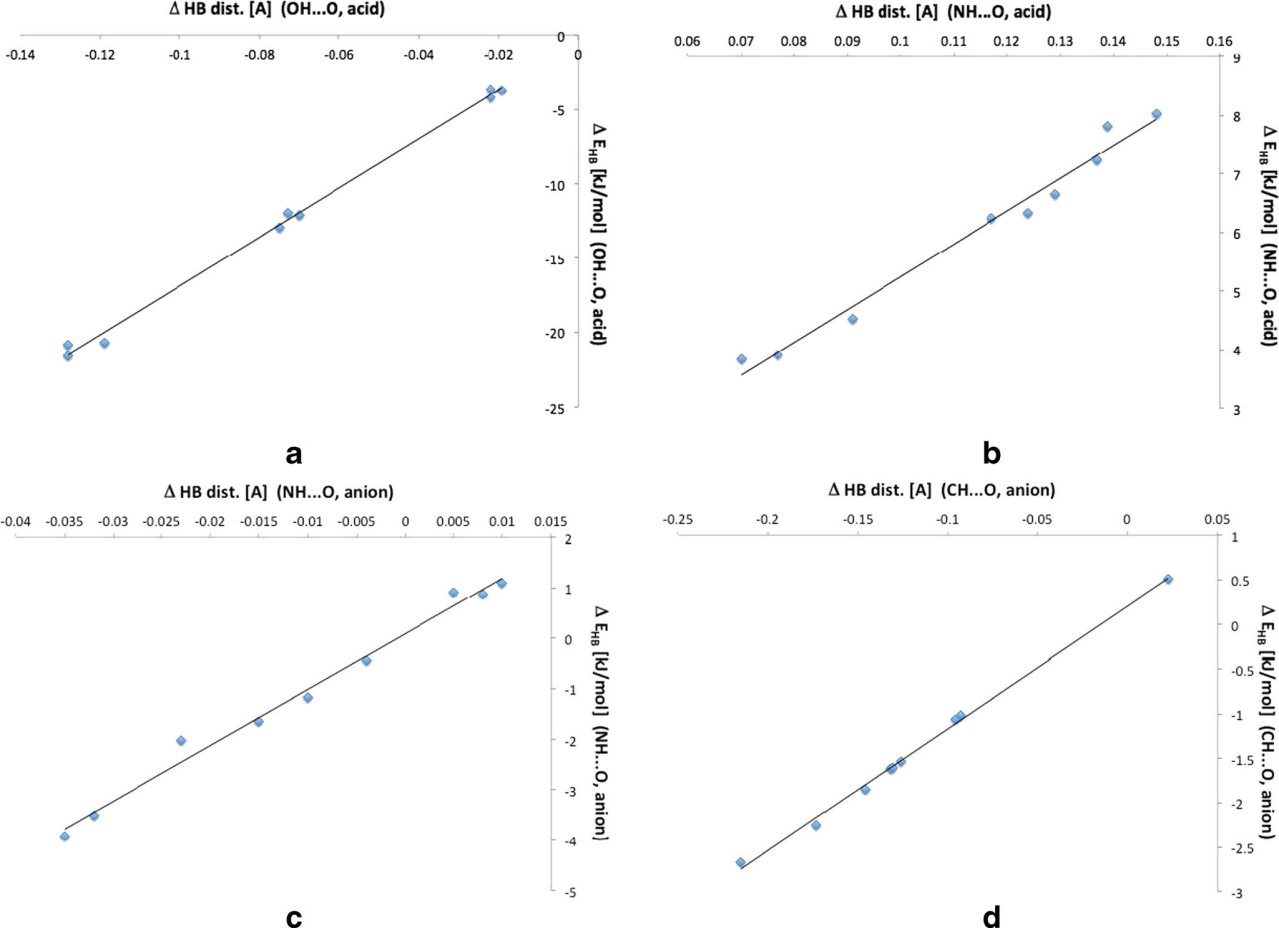
The correlation charts for changes in hydrogen bond distance (Δ HB dist.[Å]) and changes in energy of hydrogen bond (Δ E_HB_ [kJ mol^-1^])

The variability of the hydrogen bond lengths and E_HB_ calculated by Espinosa approach are linearly dependent. In Fig. 8 the correlation charts for these dependencies are shown.

For these linear functions the correlation coefficients are: *R* = 0.998 (*a*), 0.990 (*b*), 0.994 (*c*), and 0.998 (*d*). This shows that the Espinosa’s approach [49, 50] is applicable also in cases of other than originally developed for interactions (OH · · · O, NH · · · O, CH · · · F, FH · · · F, FH · · · N) [54–57]. This has also been shown for other interactions including intramolecular hydrogen bonding [58] and the negatively charged species as oxyanions [18] or π-H · · · O contacts [59].

Table 9 collects the ZPE and BSSE corrected E_int_ values for **D** complexes and the results of the reaction expressed by Eq. 4.

**Table 9:** The ZPE and BSSE corrected interaction energy (E_int_) for **D** complexes and values of ΔE_4_

R acid side	R anion side	E_int_ [kJ mol^-1^]	ΔE_4_ [kJ mol^-1^]
NO_2_	NO_2_	−83.1	−77.0
NO_2_	H	−87.3	−81.2
NO_2_	NMe_2_	−89.5	−83.3
H	NO_2_	−77.9	−73.9
H	H	−81.1	−77.0
H	NMe_2_	−82.4	−78.2
NMe_2_	NO_2_	−72.9	−70.3
NMe_2_	H	−75.7	−73.1
NMe_2_	NMe_2_	−76.5	−73.7

4 $$ \mathrm{Form}\mathbf{A}+\mathrm{anion}\to \mathrm{Form}\mathbf{C}+\mathrm{acid}+\Delta {\mathrm{E}}_4 $$

The E_int_ for **D** complexes shows that: *a*) the higher acidity of the acid determine the overall interaction in threesome complexes carrying the same acid but different anion (compare the E_int_ for the series with NO_2_-substituted acid and E_int_ with remaining acids) and *b*) the order of energy of interaction within the series carrying the same acid is determined by the character of the anion. It is worth keeping in mind that the E_int_ and ΔE_4_ in Table 9 are close to each other although those describe various properties. The E_int_ describes the intermolecular interactions in complexes **D** while ΔE_4_ include the electron reorganization and geometry relaxation upon association of an anion with complexes **A**. The charge at the formamide atoms is higher at nitrogen, oxygen, and carbon atoms than that in referring **A**-**C** complexes. The higher electron density is also observed on NH proton (acid side) of the formamide in any complex in **D** series than that in **A**. The charge is lowered at protons on the anion side (CH and NH) of the complexes **D** with respect to the values for associates **C** (SI). This suggests the charge transfer character of the interactions. These observations, however, are not the main topic of the current publication and will be used in more detailed analysis based on a larger population of complexes.

## Conclusions

The case study of formamide complexation with substituted benzoic acids and respective benzoates showed that formamide might be a molecule of choice for studying basic intermolecular interactions also in triple associates in various arrangements. The systematic changes of the substituent from electron-donating to electron-accepting revealed that in the case of acids the effect is transmitted to OH and C = O groups making them a better hydrogen bond donor and a worse hydrogen bond acceptor at a time. Opposite to that the symmetric -CO_2_^−^ moiety being a weaker base as the substituent changes to electron-accepting is a weaker hydrogen bond acceptor. In the case when both, acid and benzoate, interacts with the formamide molecule the cooperativity is observed. High correlation coefficients for properties used to describe the intermolecular interactions prove that substituent effect should be taken into account in supramolecular complexes also when weak interactions with CH group is considered. The agreement between general substituent effect and the QTAIM-derived data show this theory successfully describes intermolecular interactions of hydrogen bonded complexes of formamide and substituted benzoic acids and benzoates in this study.

## Supplement

Supporting information file contains the Cartesians for the optimized structures, their energies and values of the Laplacian and electron density at H-BCP in **D** complexes and the natural charges charts.

## Electronic supplementary material

Below is the link to the electronic supplementary material.

ESM 1 (DOC 1841 kb)
