# Supplementary material for: Substituent effects in hydrogen bonding: DFT and QTAIM studies on acids and carboxylates complexes with formamide
Source: J Mol Model. 2014 Jul 15;20(8):2356. doi: 10.1007/s00894-014-2356-8 (PMC4139586; doi:10.1007/s00894-014-2356-8)
Supplement: Supplementary file 1 — (DOC 1841 kb) [file 894_2014_2356_MOESM1_ESM.doc]

**Substituent Effects in Hydrogen Bonding: DFT and QTAIM Studies on Acids and Carboxylates Complexes with Formamide**

Borys Ośmiałowski

Faculty of Chemical Technology and Engineering, University of Technology and Life Sciences, Seminaryjna 3, PL-85-326 Bydgoszcz, Poland

**Supporting Information File**

Table of contents

Cartesian coordinates of the optimized structures S2

Values of ∇^2^*ρ* and *ρ* for complexes **D** S25

Charts and tables related to charge analysis S26

Cartesian coordinates for optimized structures

Acid R=NMe2, E(a.u.) = -554.5757842

| 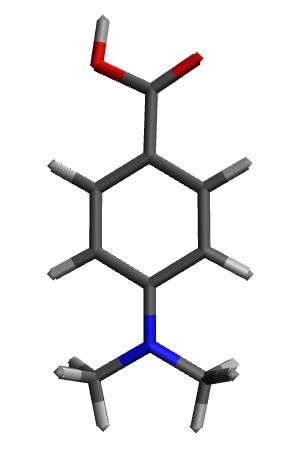 | H -4.49370 -0.94934 -0.00000  O -3.54324 -1.10858 -0.00000  C -2.94840 0.10526 -0.00000  O -3.60403 1.12460 -0.00000  C -1.48362 0.03653 -0.00000  C -0.77834 -1.17070 0.00000  C 0.59977 -1.19646 0.00000  C 1.35116 0.00178 0.00000  C 0.62960 1.21895 -0.00000  C -0.74745 1.22461 -0.00000  H -1.31970 -2.10644 0.00000  H 1.09898 -2.15316 0.00000  N 2.71472 -0.01542 0.00000  H 1.15209 2.16315 -0.00000  H -1.27805 2.16733 -0.00000  C 3.46327 1.22447 0.00000  C 3.43120 -1.27399 0.00000  H 3.24595 1.82696 0.88571  H 3.24595 1.82696 -0.88571  H 4.52514 1.00020 0.00000  H 3.19848 -1.87074 -0.88571  H 3.19848 -1.87074 0.88571  H 4.49843 -1.07705 0.00000 |
| --- | --- |

Acid R=NH2, E(a.u.) = -476.0220755

| 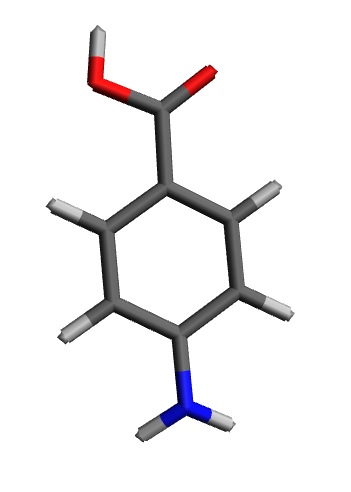 | H 3.70504 -0.93700 0.00499  O 2.75492 -1.09902 0.00284  C 2.15506 0.11067 0.00238  O 2.80213 1.13434 0.00507  C 0.68778 0.03363 -0.00150  C -0.00626 -1.18047 -0.00213  C -1.38526 -1.20904 -0.00417  C -2.12412 -0.01513 -0.00494  C -1.42726 1.20449 -0.00475  C -0.04944 1.22138 -0.00258  H 0.54296 -2.11133 0.00050  H -1.90672 -2.15827 -0.00638  N -3.49452 -0.03919 -0.05490  H -1.98112 2.13509 -0.00715  H 0.47963 2.16468 -0.00023  H -3.95388 -0.88873 0.21858  H -3.98365 0.79417 0.21683 |
| --- | --- |

Acid R=OMe, E(a.u.) = -535.1684855

| 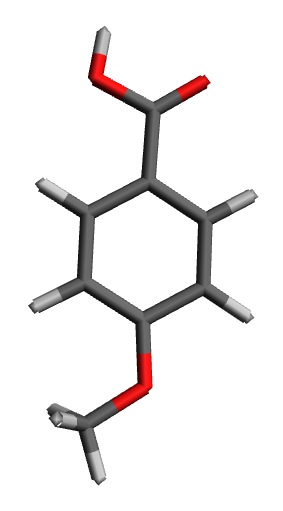 | H -4.02409 -1.22453 -0.00000  O -3.06193 -1.28542 -0.00000  C -2.58981 -0.02300 -0.00000  O -3.33138 0.93228 -0.00000  C -1.11719 0.04895 -0.00000  C -0.31086 -1.08679 0.00000  C 1.07138 -0.98333 0.00000  C 1.66786 0.27962 0.00000  C 0.86418 1.42694 0.00000  C -0.50706 1.30819 -0.00000  H -0.76513 -2.06728 0.00000  H 1.66934 -1.88200 0.00000  O 2.99503 0.49313 0.00000  H 1.34202 2.39702 0.00000  H -1.12904 2.19259 -0.00000  C 3.87734 -0.61667 0.00000  H 3.73911 -1.22950 -0.89241  H 3.73910 -1.22949 0.89242  H 4.87989 -0.20016 0.00001 |
| --- | --- |

Acid R=Me, E(a.u.) = -459.9639896

| 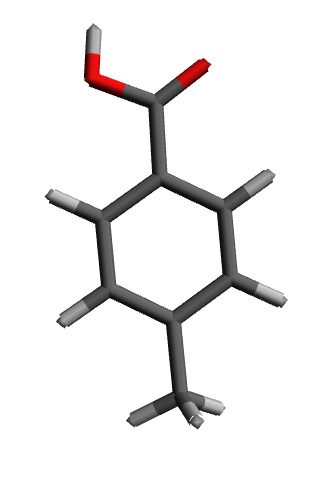 | H -3.72638 -0.94221 0.00620  O -2.77461 -1.09704 0.00367  C -2.17970 0.11018 0.00259  O -2.81792 1.13603 0.00467  C -0.70185 0.03216 -0.00072  C -0.01767 -1.18249 -0.00378  C 1.36826 -1.20136 -0.00830  C 2.10636 -0.01772 -0.00842  C 1.40976 1.19385 -0.00847  C 0.02714 1.22174 -0.00375  H -0.56934 -2.11168 -0.00507  H 1.88616 -2.15251 -0.01376  C 3.60442 -0.03732 0.01183  H 1.96161 2.12603 -0.01405  H -0.50429 2.16351 -0.00487  H 3.99748 -1.01012 -0.27871  H 3.97551 0.18413 1.01514  H 4.01909 0.71657 -0.65743 |
| --- | --- |

Acid R=H, E(a.u.) = -420.6702079

| 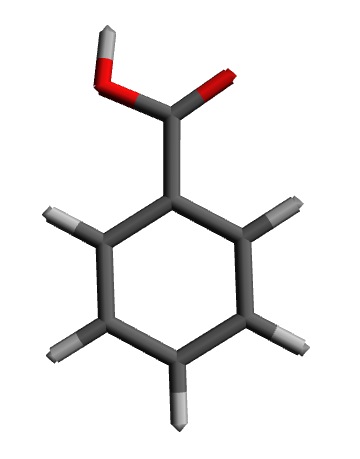 | H 3.25494 -0.92130 -0.00000  O 2.30392 -1.08159 -0.00000  C 1.70052 0.11982 -0.00000  O 2.32713 1.15179 -0.00001  C 0.21973 0.02903 0.00000  C -0.44812 -1.19595 0.00000  C -1.83474 -1.22903 0.00001  C -2.55925 -0.04278 0.00000  C -1.89710 1.18020 0.00000  C -0.51188 1.21632 -0.00000  H 0.11696 -2.11681 0.00000  H -2.35034 -2.18019 0.00001  H -3.64126 -0.07134 0.00001  H -2.46126 2.10335 -0.00000  H 0.01762 2.15896 -0.00000 |
| --- | --- |

Acid R=F, E(a.u.) = -519.920923

| 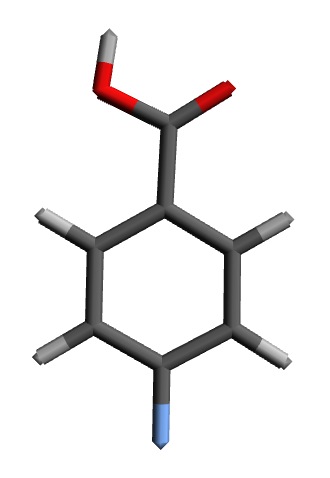 | H -3.67614 -0.94566 -0.00000  O -2.72348 -1.09606 -0.00000  C -2.13145 0.11072 0.00000  O -2.76539 1.13813 -0.00000  C -0.65220 0.03198 0.00000  C 0.02764 -1.18673 0.00000  C 1.41219 -1.21825 0.00000  C 2.09649 -0.01588 0.00000  C 1.45473 1.21042 0.00000  C 0.07121 1.22468 0.00000  H -0.52663 -2.11367 0.00000  H 1.95932 -2.15011 0.00000  F 3.43467 -0.03953 0.00000  H 2.03390 2.12271 0.00000  H -0.46317 2.16410 0.00000 |
| --- | --- |

Acid R=Cl, E(a.u.) = -880.3028111

| 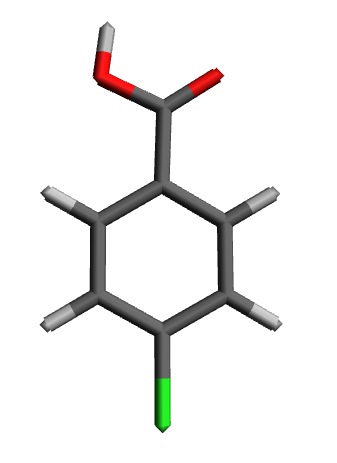 | H 1.08472 4.07426 0.00000  O 1.19952 3.11658 0.00000  C -0.02701 2.56863 0.00000  O -1.03220 3.23645 0.00000  C 0.00000 1.08585 0.00000  C 1.19274 0.36370 0.00000  C 1.17517 -1.02145 0.00000  C -0.04750 -1.67939 0.00000  C -1.24770 -0.97998 0.00000  C -1.21537 0.40351 0.00000  H 2.13988 0.88302 0.00000  H 2.09714 -1.58407 0.00000  Cl -0.07743 -3.41763 0.00000  H -2.18850 -1.51051 0.00000  H -2.13735 0.96765 0.00000 |
| --- | --- |

Acid R=CF3, E(a.u.) = -757.726852

| 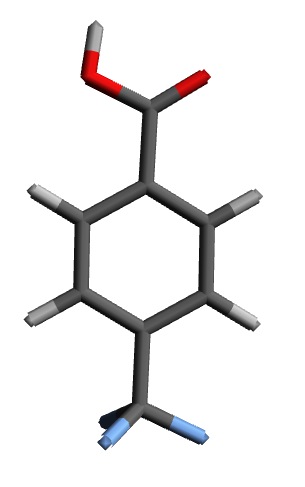 | H 4.72787 -0.98858 0.00433  O 3.77189 -1.11871 0.00232  C 3.20220 0.09551 0.00132  O 3.84493 1.11541 0.00278  C 1.71526 0.03818 -0.00154  C 1.02077 -1.17138 -0.00304  C -0.36271 -1.17444 -0.00564  C -1.05722 0.03160 -0.00719  C -0.37242 1.23935 -0.00541  C 1.01334 1.23940 -0.00289  H 1.56174 -2.10585 -0.00249  H -0.89962 -2.11336 -0.00779  C -2.55755 -0.00686 0.00005  H -0.91401 2.17381 -0.00683  H 1.56118 2.17077 -0.00213  F -3.02969 -0.58416 1.11477  F -3.03931 -0.71790 -1.02891  F -3.10675 1.20667 -0.07250 |
| --- | --- |

Acid R=NO2, E(a.u.) = -625.1563253

| 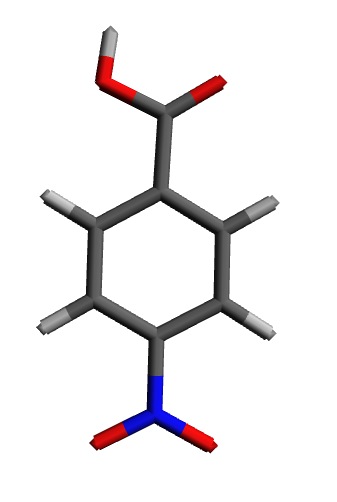 | H 4.37328 -0.96931 0.00001  O 3.41775 -1.10435 0.00000  C 2.83934 0.10379 0.00000  O 3.46797 1.13139 0.00001  C 1.35019 0.03245 0.00000  C 0.67027 -1.18468 0.00000  C -0.71419 -1.20620 -0.00000  C -1.39471 0.00029 -0.00000  C -0.74282 1.22331 0.00000  C 0.64047 1.23179 0.00000  H 1.22114 -2.11307 0.00000  H -1.26160 -2.13582 -0.00000  N -2.87655 -0.01746 -0.00000  H -1.31232 2.13962 0.00000  H 1.18236 2.16631 0.00000  O -3.42761 -1.09914 -0.00001  O -3.45291 1.05085 -0.00000 |
| --- | --- |

Anion R=NMe2, E(a.u.) = -554.0854922

| 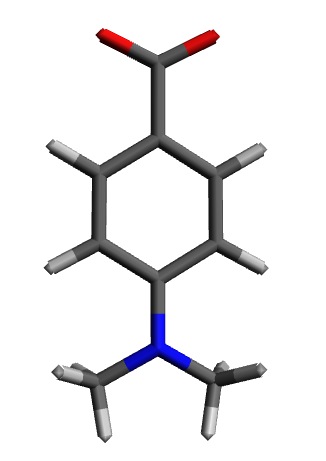 | O -3.63003 1.12064 0.10779  C -3.07230 0.00120 0.02591  O -3.63657 -1.11650 -0.02999  C -1.54559 -0.00071 -0.01058  C -0.81493 1.18424 -0.03244  C 0.57101 1.19741 -0.06735  C 1.30735 -0.00235 -0.08580  C 0.57021 -1.20097 -0.04012  C -0.81584 -1.18657 -0.01316  H -1.35961 2.11963 -0.01605  H 1.07763 2.15149 -0.07944  N 2.69427 -0.00399 -0.16349  H 1.07636 -2.15535 -0.02900  H -1.36042 -2.12201 0.01306  C 3.41206 -1.22625 0.12008  H 3.10873 -2.02633 -0.55524  H 3.26491 -1.57656 1.15041  H 4.47512 -1.05810 -0.03410  C 3.41182 1.23053 0.06024  H 3.27177 1.62730 1.07462  H 3.10163 1.99838 -0.64865  H 4.47402 1.05714 -0.09430 |
| --- | --- |

Anion R=NH2, E(a.u.) = -475.5334911

| 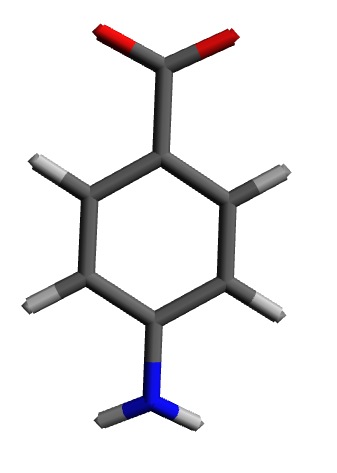 | O -2.83382 1.11966 0.06892  C -2.27484 0.00024 0.00489  O -2.83554 -1.11865 -0.05227  C -0.74589 -0.00029 -0.00369  C -0.02081 1.18966 -0.02834  C 1.36588 1.19931 -0.03616  C 2.08346 -0.00034 -0.00946  C 1.36593 -1.20004 0.01625  C -0.02082 -1.19035 0.01616  H -0.57096 2.12165 -0.03956  H 1.90353 2.14123 -0.06143  N 3.47897 -0.00157 -0.06776  H 1.90362 -2.14209 0.03419  H -0.57064 -2.12249 0.03463  H 3.90938 0.83551 0.28743  H 3.90978 -0.82003 0.32798 |
| --- | --- |

Anion R=OMe, E(a.u.) = -534.6825722

| 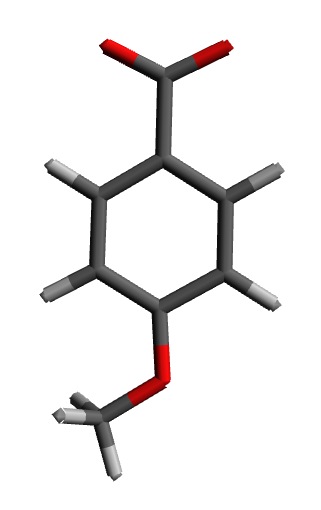 | O -3.36389 0.91884 0.00001  C -2.69993 -0.14264 0.00000  O -3.14583 -1.31262 -0.00001  C -1.17578 0.00812 -0.00000  C -0.57332 1.26811 -0.00001  C 0.80243 1.41363 -0.00001  C 1.62442 0.28460 -0.00000  C 1.04649 -0.98307 0.00000  C -0.34062 -1.10217 -0.00000  H -1.21283 2.14082 -0.00001  H 1.26339 2.39357 -0.00001  O 2.96446 0.51972 -0.00000  H 1.65734 -1.87462 0.00000  H -0.79533 -2.08410 0.00000  C 3.84544 -0.58212 0.00001  H 3.71223 -1.20115 -0.89074  H 3.71222 -1.20114 0.89076  H 4.85013 -0.16760 0.00001 |
| --- | --- |

Anion R=Me, E(a.u.) = -459.4791172

| 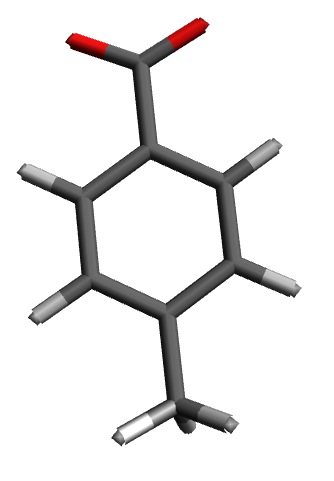 | O -2.84913 -1.11559 -0.05107  C -2.29524 -0.00059 0.00386  O -2.85062 1.11344 0.06244  C -0.76230 0.00055 -0.00077  C -0.04144 -1.19183 0.01678  C 1.34665 -1.19247 0.01410  C 2.06833 0.00211 -0.01125  C 1.34579 1.19470 -0.03556  C -0.04357 1.19264 -0.02622  H -0.59339 -2.12258 0.03016  H 1.88156 -2.13653 0.02809  C 3.56976 -0.00075 0.01630  H 1.87942 2.13897 -0.06273  H -0.59642 2.12279 -0.04223  H 3.97875 -0.81718 -0.58017  H 3.94137 -0.12970 1.03620  H 3.97885 0.93525 -0.36375 |
| --- | --- |

Anion R=H, E(a.u.) = -420.1870492

| 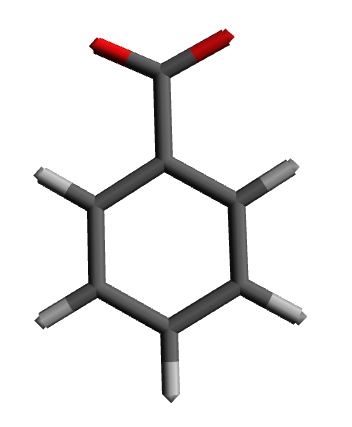 | O -2.36806 1.11902 0.07012  C -1.81330 0.00000 0.00000  O -2.36806 -1.11902 -0.07012  C -0.27699 0.00000 0.00000  C 0.43713 1.19660 -0.02670  C 1.82689 1.20120 -0.03086  C 2.52723 0.00000 0.00000  C 1.82689 -1.20120 0.03086  C 0.43713 -1.19660 0.02670  H -0.12139 2.12309 -0.04388  H 2.36563 2.14124 -0.05627  H 3.61068 0.00000 0.00000  H 2.36563 -2.14124 0.05627  H -0.12139 -2.12309 0.04388 |
| --- | --- |

Anion R=F, E(a.u.) = -519.4396157

| 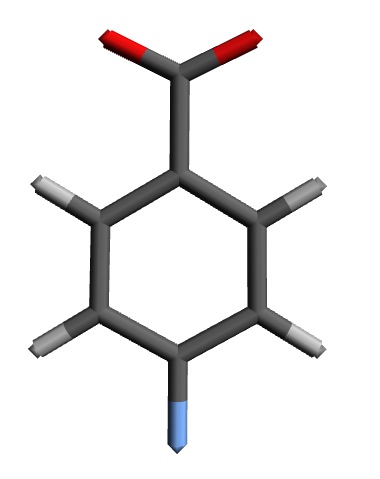 | O -2.80001 1.11994 0.06294  C -2.24717 0.00000 0.00000  O -2.80001 -1.11994 -0.06294  C -0.71193 0.00000 0.00000  C 0.00247 1.19589 -0.02394  C 1.39165 1.21070 -0.02768  C 2.05696 0.00000 0.00000  C 1.39165 -1.21070 0.02768  C 0.00247 -1.19589 0.02394  H -0.55350 2.12340 -0.03971  H 1.95413 2.13469 -0.05002  F 3.40914 0.00000 0.00000  H 1.95413 -2.13469 0.05002  H -0.55350 -2.12340 0.03971 |
| --- | --- |

Anion R=Cl, E(a.u.) = -879.822748

| 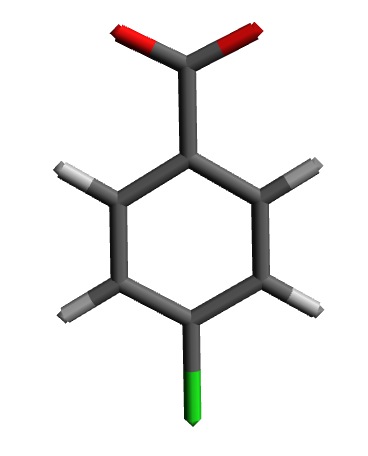 | O -3.23443 1.12018 0.06425  C -2.68343 0.00000 0.00000  O -3.23443 -1.12018 -0.06425  C -1.14717 0.00000 0.00000  C -0.43223 1.19482 -0.02436  C 0.95637 1.20891 -0.02831  C 1.63566 0.00000 0.00000  C 0.95637 -1.20891 0.02831  C -0.43223 -1.19482 0.02436  H -0.98612 2.12375 -0.04030  H 1.50349 2.14129 -0.05144  Cl 3.38801 0.00000 0.00000  H 1.50349 -2.14129 0.05144  H -0.98612 -2.12374 0.04030 |
| --- | --- |

Anion R=CF3, E(a.u.) = -757.2495076

| 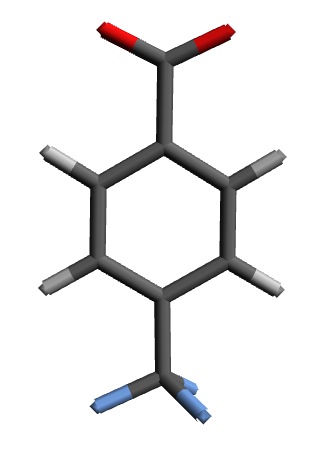 | O -3.85195 -1.13587 -0.06541  C -3.31673 -0.00910 0.00123  O -3.87779 1.10478 0.06891  C -1.77775 0.00668 -0.00052  C -1.05561 -1.18569 0.02382  C 0.32907 -1.18219 0.02478  C 1.01742 0.02880 -0.00658  C 0.31344 1.22632 -0.03381  C -1.07500 1.20708 -0.02735  H -1.60642 -2.11580 0.04140  H 0.87533 -2.11705 0.04709  C 2.51084 0.00548 -0.00034  H 0.84422 2.16806 -0.05922  H -1.63739 2.13048 -0.04269  F 3.01335 -0.71227 -1.02156  F 3.00546 -0.55596 1.11898  F 3.05765 1.22365 -0.08655 |
| --- | --- |

Anion R=NO2, E(a.u.) = -624.6826147

| 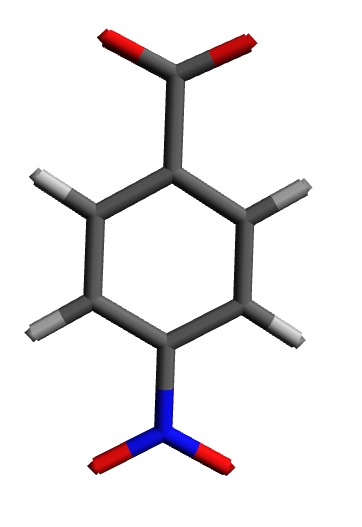 | O 3.49586 1.11953 -0.09063  C 2.95181 0.00000 0.00000  O 3.49586 -1.11953 0.09063  C 1.41085 0.00000 0.00000  C 0.70137 1.20063 0.01386  C -0.68279 1.21355 0.01898  C -1.35826 0.00000 0.00000  C -0.68279 -1.21355 -0.01898  C 0.70137 -1.20063 -0.01386  H 1.26078 2.12538 0.01968  H -1.23916 2.13862 0.03397  N -2.82700 0.00000 0.00000  H -1.23916 -2.13862 -0.03397  H 1.26078 -2.12538 -0.01968  O -3.40233 1.07277 0.05352  O -3.40233 -1.07277 -0.05352 |
| --- | --- |

Formamide, E(a.u.) = -169.8633318

| 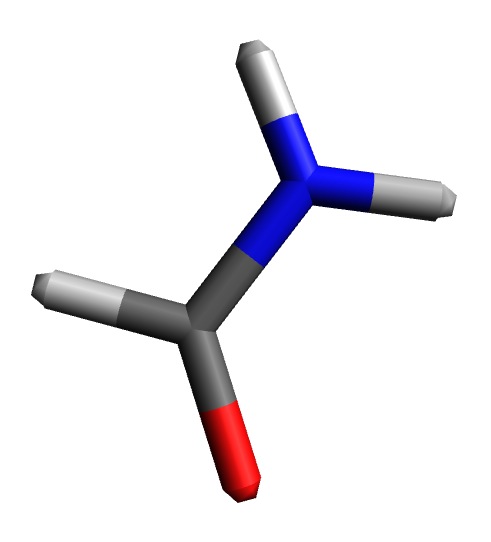 | O -1.19553 -0.24270 -0.00002  C -0.15462 0.38367 -0.00000  N 1.07703 -0.15838 0.00001  H -0.13003 1.48604 0.00000  H 1.89607 0.41941 0.00003  H 1.18670 -1.15717 0.00001 |
| --- | --- |

Form **A** R=NMe_2_, E(a.u.) = -724.456733

| 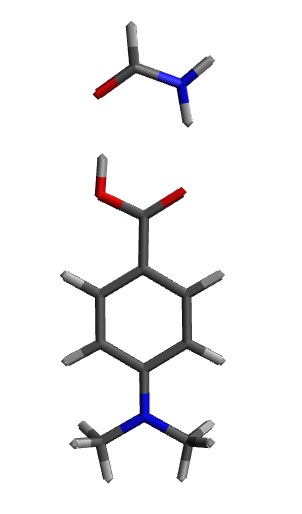 | O -4.83828 -1.14570 -0.00000  C -5.54922 -0.14521 0.00000  N -5.10157 1.10958 0.00001  H -6.64518 -0.23197 0.00001  H -5.74578 1.87742 0.00001  H -4.09917 1.27912 0.00000  H -3.10161 -1.06473 -0.00000  O -2.11753 -1.14957 -0.00000  C -1.54422 0.04923 -0.00000  O -2.18971 1.08750 -0.00001  C -0.07470 0.00888 -0.00000  C 0.64633 -1.18850 0.00000  C 2.02508 -1.19749 0.00000  C 2.76136 0.00969 0.00000  C 2.02461 1.21719 -0.00000  C 0.64689 1.20515 -0.00000  H 0.11446 -2.12969 0.00000  H 2.53611 -2.14804 0.00000  N 4.12605 0.00994 0.00000  H 2.53521 2.16798 -0.00000  H 0.10487 2.14133 -0.00000  C 4.85802 1.25917 0.00000  C 4.85836 -1.23913 0.00000  H 4.63301 1.85920 0.88562  H 4.63302 1.85919 -0.88562  H 5.92282 1.04902 0.00001  H 4.63339 -1.83918 -0.88559  H 4.63339 -1.83918 0.88560  H 5.92312 -1.02875 0.00000 |
| --- | --- |

Form **A** R=NH_2_, E(a.u.) =-645.9031847

| 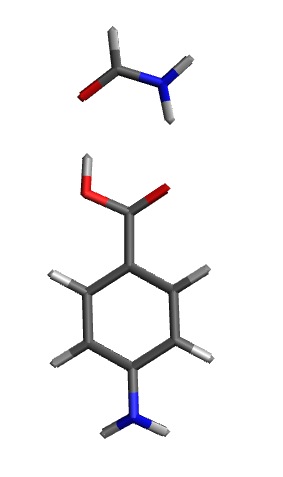 | O 4.00045 -1.14833 0.00588  C 4.71650 -0.15140 0.00068  N 4.27551 1.10566 -0.00291  H 5.81186 -0.24399 -0.00137  H 4.92369 1.87016 -0.00769  H 3.27440 1.28082 -0.00140  H 2.27085 -1.06530 0.00390  O 1.28576 -1.14741 0.00194  C 0.71530 0.05102 0.00157  O 1.35897 1.08930 0.00396  C -0.75736 0.01178 -0.00192  C -1.47430 -1.18839 -0.00440  C -2.85402 -1.19193 -0.00633  C -3.57066 0.01490 -0.00490  C -2.85152 1.22090 -0.00276  C -1.47286 1.21205 -0.00089  H -0.93991 -2.12786 -0.00334  H -3.39273 -2.13156 -0.00997  N -4.94307 0.01655 -0.05595  H -3.38835 2.16163 -0.00365  H -0.92667 2.14551 0.00291  H -5.41507 -0.82404 0.22424  H -5.41311 0.85704 0.22781 |
| --- | --- |

Form **A** R=OMe, E(a.u.) = -705.0498178

| 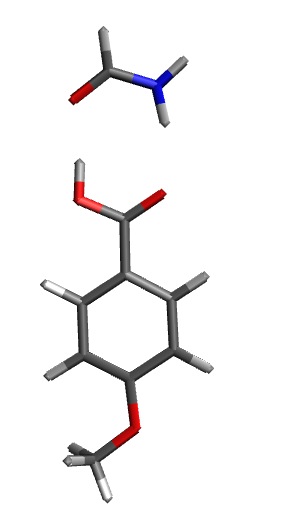 | O -4.37258 -1.26486 -0.00000  C -5.14163 -0.30798 0.00000  N -4.76996 0.97113 0.00001  H -6.23002 -0.46041 0.00000  H -5.45893 1.69924 0.00000  H -3.78054 1.20135 0.00000  H -2.66122 -1.09859 -0.00000  O -1.67186 -1.13105 -0.00000  C -1.16115 0.09138 -0.00000  O -1.84790 1.09976 -0.00001  C 0.31677 0.12196 -0.00000  C 1.08262 -1.04098 -0.00000  C 2.46809 -0.98802 0.00000  C 3.10965 0.25240 0.00000  C 2.34762 1.42738 -0.00000  C 0.97243 1.35762 -0.00000  H 0.59095 -2.00325 -0.00000  H 3.03322 -1.90783 0.00000  O 4.44469 0.41866 0.00000  H 2.85999 2.37982 -0.00000  H 0.38299 2.26400 -0.00000  C 5.28568 -0.72208 0.00000  H 5.12545 -1.32988 -0.89229  H 5.12544 -1.32987 0.89230  H 6.30294 -0.34263 0.00001 |
| --- | --- |

Form **A** R=Me, E(a.u.) = -629.8453774

| 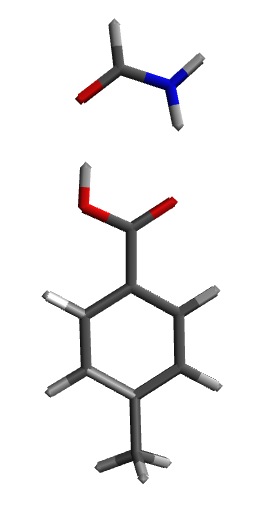 | O -4.00237 -1.14722 0.00403  C -4.72783 -0.15687 0.00360  N -4.29937 1.10434 0.00208  H -5.82190 -0.26043 0.00449  H -4.95490 1.86265 0.00180  H -3.30103 1.29037 0.00126  H -2.28939 -1.06526 0.00180  O -1.30185 -1.14324 0.00034  C -0.73543 0.05233 -0.00077  O -1.37038 1.09276 0.00008  C 0.74773 0.01168 -0.00251  C 1.45497 -1.18986 -0.00587  C 2.84076 -1.18425 -0.00976  C 3.55792 0.01278 -0.00854  C 2.83943 1.21071 -0.00865  C 1.45560 1.21301 -0.00472  H 0.91761 -2.12744 -0.00849  H 3.37574 -2.12611 -0.01623  C 5.05628 0.01677 0.01739  H 3.37412 2.15291 -0.01423  H 0.90748 2.14515 -0.00638  H 5.46657 -0.90727 -0.38730  H 5.41952 0.11564 1.04315  H 5.46198 0.85324 -0.55098 |
| --- | --- |

Form **A** R=H, E(a.u.) = -590.551759

| 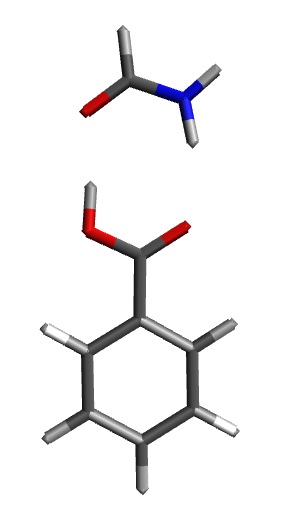 | O 3.51482 -1.15014 -0.00000  C 4.24515 -0.16327 0.00000  N 3.82313 1.10004 0.00001  H 5.33856 -0.27245 0.00001  H 4.48250 1.85504 0.00001  H 2.82609 1.29151 0.00000  H 1.80842 -1.06577 -0.00001  O 0.81986 -1.14094 -0.00001  C 0.25642 0.05445 -0.00000  O 0.88988 1.09483 -0.00000  C -1.23048 0.01537 -0.00000  C -1.93264 -1.18994 0.00000  C -3.31981 -1.18568 0.00001  C -4.01205 0.01978 0.00000  C -3.31616 1.22384 -0.00000  C -1.93009 1.22143 -0.00000  H -1.39041 -2.12448 0.00000  H -3.86121 -2.12253 0.00001  H -5.09451 0.02115 0.00000  H -3.85475 2.16230 -0.00000  H -1.37500 2.14921 -0.00001 |
| --- | --- |

Form **A** R=F, E(a.u.) = -689.8026249

| 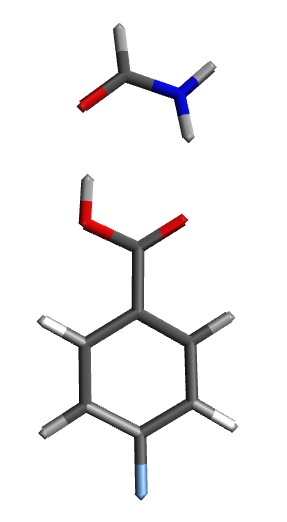 | O 3.95488 -1.14810 -0.00000  C 4.68680 -0.16228 -0.00000  N 4.26687 1.10164 0.00000  H 5.77991 -0.27340 -0.00000  H 4.92750 1.85556 0.00000  H 3.27036 1.29502 0.00000  H 2.25350 -1.06530 0.00000  O 1.26436 -1.14030 0.00000  C 0.70052 0.05450 0.00000  O 1.33119 1.09655 0.00000  C -0.78418 0.01309 0.00000  C -1.48642 -1.19248 0.00000  C -2.87159 -1.20017 0.00000  C -3.53461 0.01391 0.00000  C -2.87106 1.22832 -0.00000  C -1.48701 1.21755 0.00000  H -0.94598 -2.12760 0.00000  H -3.43502 -2.12237 0.00000  F -4.87404 0.01431 -0.00000  H -3.43405 2.15080 -0.00000  H -0.93606 2.14731 -0.00000 |
| --- | --- |

Form **A** R=Cl, E(a.u.) = -1050.184566

| 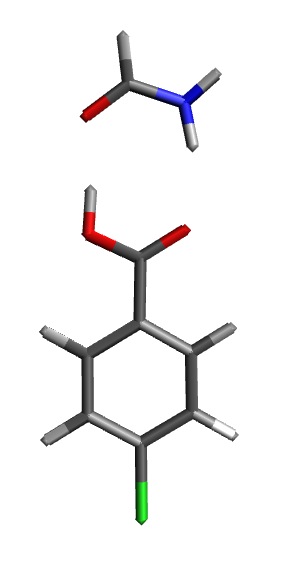 | O 4.39670 -1.14665 0.00000  C 5.13015 -0.16190 0.00000  N 4.71235 1.10267 0.00000  H 6.22301 -0.27487 0.00000  H 5.37423 1.85552 0.00000  H 3.71638 1.29798 0.00000  H 2.69904 -1.06511 0.00000  O 1.70933 -1.13961 -0.00000  C 1.14593 0.05443 0.00000  O 1.77397 1.09756 0.00000  C -0.34046 0.01199 0.00000  C -1.04243 -1.19243 0.00000  C -2.42788 -1.19983 0.00000  C -3.10719 0.01095 0.00000  C -2.42894 1.22301 0.00000  C -1.04464 1.21437 0.00000  H -0.50373 -2.12872 0.00000  H -2.97415 -2.13168 0.00000  Cl -4.84681 0.01022 0.00000  H -2.97607 2.15436 0.00000  H -0.49667 2.14607 0.00000 |
| --- | --- |

Form **A** R=CF_3_, E(a.u.) = -927.6088118

| 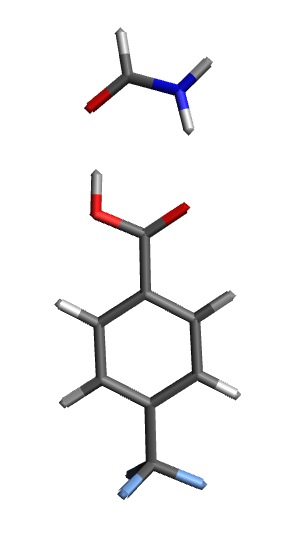 | O 5.07962 -1.15575 0.00173  C 5.82317 -0.17843 0.00577  N 5.41877 1.09035 0.00650  H 6.91457 -0.30307 0.00915  H 6.08853 1.83625 0.01008  H 4.42547 1.29687 0.00350  H 3.39238 -1.06556 -0.00190  O 2.40081 -1.13285 -0.00341  C 1.84515 0.06246 -0.00394  O 2.47304 1.10424 -0.00312  C 0.35357 0.02537 -0.00533  C -0.34917 -1.17914 -0.00488  C -1.73273 -1.17469 -0.00567  C -2.42054 0.03530 -0.00748  C -1.72807 1.23869 -0.00772  C -0.34203 1.22992 -0.00697  H 0.18899 -2.11529 -0.00413  H -2.27514 -2.11056 -0.00623  C -3.92052 0.00598 0.00223  H -2.26412 2.17645 -0.00936  H 0.21139 2.15796 -0.00776  F -4.39563 -0.56459 1.11955  F -4.40947 -0.70528 -1.02354  F -4.46314 1.22281 -0.07340 |
| --- | --- |

Form **A** R=NO_2_, E(a.u.) = -795.0385881

| 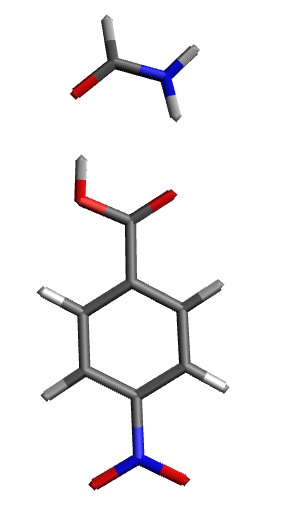 | O -4.66799 -1.14614 -0.00001  C -5.41258 -0.16938 0.00000  N -5.00987 1.09978 0.00002  H -6.50361 -0.29556 0.00001  H -5.68057 1.84488 0.00003  H -4.01741 1.30828 0.00002  H -2.99062 -1.06461 -0.00001  O -1.99775 -1.13438 -0.00001  C -1.43865 0.05724 -0.00001  O -2.05691 1.10379 -0.00000  C 0.05571 0.01285 -0.00000  C 0.74943 -1.19626 -0.00000  C 2.13406 -1.20402 0.00000  C 2.80260 0.00946 0.00000  C 2.13723 1.22539 -0.00000  C 0.75376 1.21873 -0.00000  H 0.20547 -2.12876 -0.00000  H 2.69116 -2.12796 0.00000  N 4.28360 0.00740 0.00000  H 2.69682 2.14780 0.00000  H 0.20198 2.14741 -0.00000  O 4.84698 -1.06820 0.00003  O 4.84958 1.08172 -0.00002 |
| --- | --- |

Form **B** R=NMe2, E(a.u.) = -724.452479

| 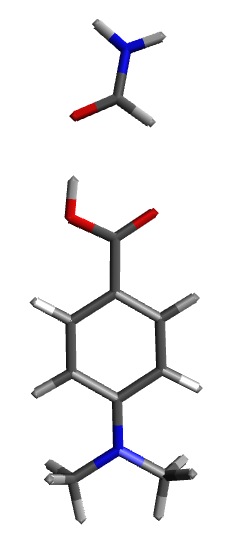 | O 4.73240 -0.93220 -0.00004  C 5.19766 0.20094 0.00004  N 6.50560 0.47647 0.00007  H 4.54706 1.08514 0.00009  H 6.83118 1.42446 0.00014  H 7.17984 -0.26888 0.00003  H 3.00229 -1.01578 -0.00005  O 2.02124 -1.12580 -0.00004  C 1.44533 0.07689 -0.00003  O 2.09289 1.10840 -0.00004  C -0.02738 0.02901 -0.00002  C -0.74503 -1.16978 -0.00001  C -2.12450 -1.18328 0.00001  C -2.86441 0.02105 0.00000  C -2.13140 1.23030 -0.00002  C -0.75299 1.22219 -0.00003  H -0.21033 -2.10941 -0.00000  H -2.63246 -2.13556 0.00002  N -4.23026 0.01694 0.00002  H -2.64479 2.17969 -0.00002  H -0.21311 2.15975 -0.00004  C -4.96573 1.26360 0.00001  H -4.74273 1.86476 -0.88552  H -4.74272 1.86477 0.88553  H -6.03001 1.05042 0.00001  C -4.95791 -1.23432 0.00005  H -4.73103 -1.83405 0.88555  H -4.73105 -1.83408 -0.88545  H -6.02352 -1.02787 0.00006 |
| --- | --- |

Form **B** R=NH_2_, E(a.u.) = -645.8990599

| 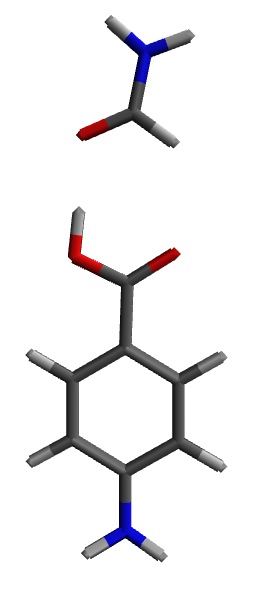 | O -3.87795 -0.93244 0.00828  C -4.35130 0.19744 -0.00217  N -5.66096 0.46328 -0.00684  H -3.70730 1.08641 -0.00832  H -5.99369 1.40873 -0.01633  H -6.32962 -0.28708 -0.00240  H -2.15447 -1.01330 0.00732  O -1.17243 -1.12121 0.00489  C -0.59872 0.08078 0.00453  O -1.24391 1.11275 0.00805  C 0.87701 0.03345 -0.00054  C 1.59027 -1.16832 -0.00426  C 2.97073 -1.17654 -0.00781  C 3.69122 0.02728 -0.00679  C 2.97610 1.23503 -0.00342  C 1.59679 1.23056 0.00009  H 1.05285 -2.10611 -0.00291  H 3.50625 -2.11812 -0.01246  N 5.06555 0.02438 -0.06082  H 3.51587 2.17419 -0.00465  H 1.05293 2.16551 0.00487  H 5.53146 -0.81669 0.22862  H 5.53518 0.86205 0.23245 |
| --- | --- |

Form **B** R=OMe, E(a.u.) = -705.0458497

| 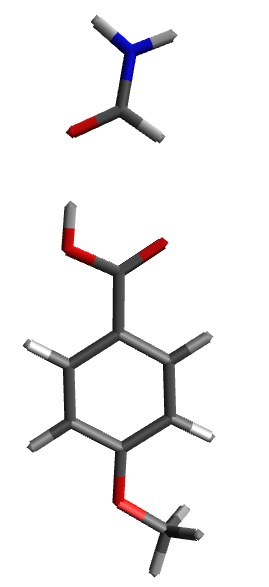 | O 4.35919 0.83598 0.00001  C 4.78332 -0.31365 -0.00000  N 6.07970 -0.63628 -0.00001  H 4.10140 -1.17376 -0.00001  H 6.36998 -1.59579 -0.00002  H 6.78085 0.08389 0.00000  H 2.65230 0.99320 0.00001  O 1.67398 1.14144 0.00001  C 1.04901 -0.03183 0.00000  O 1.64116 -1.09369 0.00000  C -0.42776 0.08344 0.00000  C -1.08447 1.31892 -0.00000  C -2.46149 1.38458 -0.00001  C -3.22196 0.20966 -0.00000  C -2.57868 -1.03010 0.00000  C -1.19369 -1.07823 0.00000  H -0.50611 2.23169 -0.00001  H -2.97503 2.33648 -0.00001  O -4.55841 0.37399 -0.00000  H -3.14227 -1.95095 0.00001  H -0.68963 -2.03498 0.00001  C -5.39632 -0.76836 0.00000  H -5.23494 -1.37620 0.89220  H -5.23494 -1.37621 -0.89219  H -6.41470 -0.39173 -0.00000 |
| --- | --- |

Form **B** R=Me, E(a.u.) = -629.8415791

| 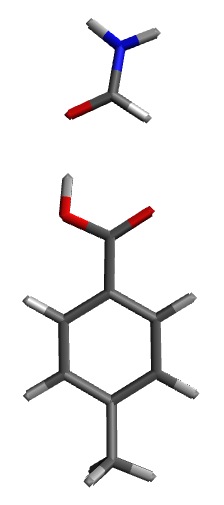 | O -3.88096 -0.92756 0.00001  C -4.36965 0.19617 -0.00000  N -5.68231 0.44344 -0.00002  H -3.73850 1.09430 -0.00000  H -6.02872 1.38401 -0.00002  H -6.34010 -0.31650 -0.00002  H -2.17290 -1.00547 0.00001  O -1.18850 -1.11003 0.00001  C -0.61770 0.08873 0.00001  O -1.25383 1.12349 0.00001  C 0.86775 0.03887 0.00000  C 1.57138 -1.16664 -0.00000  C 2.95562 -1.16710 -0.00001  C 3.67918 0.02813 -0.00001  C 2.96661 1.22714 -0.00000  C 1.58052 1.23496 0.00000  H 1.02957 -2.10170 -0.00001  H 3.48715 -2.11146 -0.00002  C 5.17805 0.00997 -0.00000  H 3.50475 2.16713 -0.00000  H 1.03615 2.16944 0.00001  H 5.56088 -0.51453 -0.87726  H 5.56087 -0.51427 0.87741  H 5.59283 1.01641 -0.00015 |
| --- | --- |

Form **B** R=H, E(a.u.) = -590.5480977

| 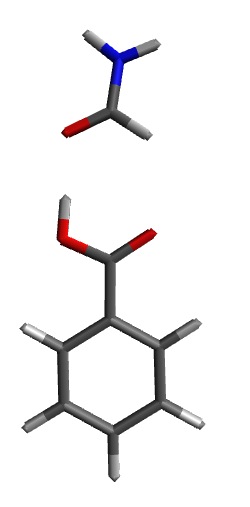 | O 3.38501 -0.92558 -0.00002  C 3.87807 0.19639 0.00001  N 5.19148 0.43831 0.00002  H 3.25060 1.09705 0.00003  H 5.54176 1.37746 0.00004  H 5.84616 -0.32433 0.00001  H 1.68300 -1.00367 -0.00002  O 0.69778 -1.10765 -0.00001  C 0.12724 0.08961 -0.00001  O 0.75956 1.12588 -0.00001  C -1.36163 0.03865 -0.00000  C -2.05761 -1.17009 0.00001  C -3.44510 -1.17402 0.00001  C -4.14458 0.02718 0.00001  C -3.45511 1.23493 -0.00000  C -2.06884 1.24006 -0.00001  H -1.51011 -2.10160 0.00001  H -3.98101 -2.11414 0.00002  H -5.22708 0.02233 0.00002  H -3.99888 2.17051 -0.00000  H -1.51827 2.17068 -0.00002 |
| --- | --- |

Form **B** R=F, E(a.u.) = -689.7990467

| 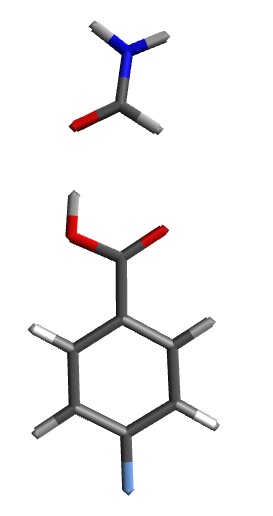 | O 3.83636 -0.92268 -0.00002  C 4.33211 0.19825 0.00001  N 5.64589 0.43685 0.00003  H 3.70685 1.10043 0.00002  H 5.99858 1.37510 0.00005  H 6.29863 -0.32746 0.00002  H 2.13957 -1.00141 -0.00002  O 1.15378 -1.10552 -0.00002  C 0.58232 0.09082 -0.00002  O 1.21124 1.12912 -0.00002  C -0.90440 0.03683 -0.00001  C -1.59984 -1.17245 0.00000  C -2.98536 -1.18890 0.00001  C -3.65592 0.02078 0.00001  C -2.99965 1.23896 -0.00000  C -1.61524 1.23630 -0.00001  H -1.05373 -2.10431 0.00001  H -3.54303 -2.11469 0.00002  F -4.99620 0.01293 0.00002  H -3.56812 2.15814 -0.00000  H -1.06921 2.16909 -0.00002 |
| --- | --- |

Form **B** R=Cl, E(a.u.) = -1050.18108

| 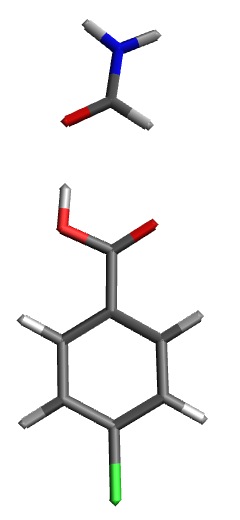 | O -4.28746 -0.92098 0.00000  C -4.78616 0.19874 0.00000  N -6.10043 0.43374 0.00000  H -4.16339 1.10263 -0.00000  H -6.45571 1.37101 0.00001  H -6.75105 -0.33239 0.00001  H -2.59451 -0.99956 -0.00000  O -1.60813 -1.10312 -0.00000  C -1.03693 0.09235 -0.00000  O -1.66300 1.13189 -0.00000  C 0.45139 0.03708 -0.00000  C 1.14635 -1.17120 -0.00000  C 2.53211 -1.18767 0.00000  C 3.21902 0.01856 0.00000  C 2.54839 1.23468 0.00000  C 1.16379 1.23438 -0.00000  H 0.60179 -2.10413 -0.00000  H 3.07248 -2.12303 0.00000  Cl 4.95953 0.00668 0.00000  H 3.10136 2.16264 0.00000  H 0.62091 2.16918 -0.00000 |
| --- | --- |

Form **B** R=CF_3_, E(a.u.) = -927.6054962

| 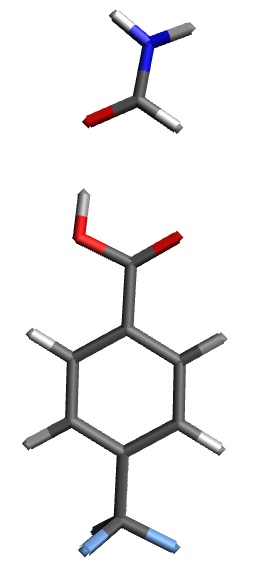 | O 4.98753 -0.92539 0.00076  C 5.50040 0.18814 0.00448  N 6.81719 0.40603 0.00933  H 4.88955 1.10008 0.00405  H 7.18463 1.33862 0.01217  H 7.45771 -0.36859 0.01011  H 3.30367 -0.99291 -0.00312  O 2.31537 -1.09028 -0.00446  C 1.75045 0.10577 -0.00497  O 2.37513 1.14488 -0.00450  C 0.25725 0.05416 -0.00583  C -0.43719 -1.15511 -0.00547  C -1.82094 -1.16129 -0.00575  C -2.51814 0.04330 -0.00690  C -1.83428 1.25168 -0.00707  C -0.44810 1.25290 -0.00685  H 0.10797 -2.08722 -0.00519  H -2.35629 -2.10131 -0.00633  C -4.01741 0.00245 0.00285  H -2.37720 2.18554 -0.00818  H 0.09919 2.18470 -0.00756  F -4.48893 -0.57668 1.11765  F -4.50180 -0.70829 -1.02591  F -4.56994 1.21532 -0.06758 |
| --- | --- |

Form **B** R=NO_2_, E(a.u.) = -795.0354717

| 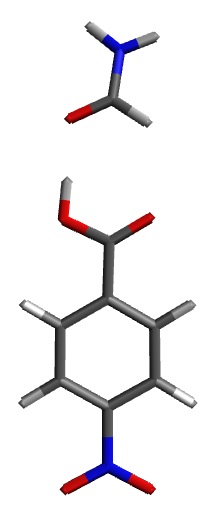 | O 4.56651 -0.91538 -0.00001  C 5.08197 0.19728 0.00000  N 6.39884 0.41181 0.00001  H 4.47337 1.11065 0.00000  H 6.76866 1.34349 0.00002  H 7.03743 -0.36444 0.00001  H 2.89383 -0.98911 -0.00001  O 1.90427 -1.08924 -0.00000  C 1.33482 0.10245 -0.00000  O 1.94867 1.14714 -0.00000  C -0.16088 0.04216 -0.00000  C -0.84515 -1.17232 0.00000  C -2.22976 -1.19207 0.00000  C -2.90898 0.01558 0.00000  C -2.25333 1.23698 -0.00000  C -0.86983 1.24164 -0.00000  H -0.29332 -2.10020 0.00001  H -2.77870 -2.12090 0.00001  N -4.38920 0.00107 0.00000  H -2.82073 2.15469 -0.00001  H -0.32500 2.17454 -0.00001  O -4.94419 -1.07917 0.00000  O -4.96479 1.07052 0.00000 |
| --- | --- |

Form **C** R=NMe2, E(a.u.) = -723.9668633

| 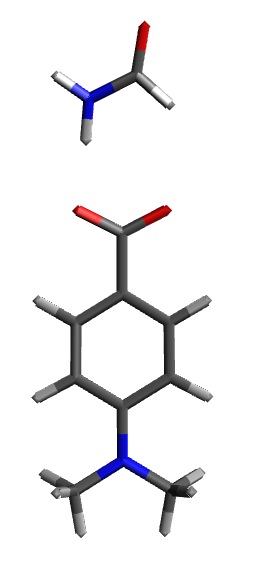 | O 6.69473 -0.54018 0.12215  C 5.48416 -0.34406 0.07579  N 4.88633 0.84751 0.00319  H 4.75363 -1.16751 0.08918  H 3.86094 0.92821 -0.02762  H 5.45957 1.67289 -0.01372  O 2.09560 -1.10609 -0.04857  C 1.51349 -0.00076 -0.05614  O 2.07425 1.12971 -0.06223  C -0.00533 -0.00434 -0.05864  C -0.72813 -1.19463 -0.06925  C -2.11348 -1.21462 -0.06965  C -2.85647 -0.01791 -0.06929  C -2.12530 1.18547 -0.03859  C -0.73971 1.17870 -0.04001  H -0.17933 -2.12791 -0.07225  H -2.61575 -2.17091 -0.07138  N -4.24175 -0.02397 -0.11334  H -2.63660 2.13667 -0.01524  H -0.20040 2.11696 -0.02043  C -4.96267 1.20140 0.14762  H -4.67551 1.98360 -0.55557  H -4.80010 1.58159 1.16456  H -6.02694 1.02249 0.01702  C -4.95136 -1.26126 0.12139  H -4.78453 -1.66172 1.12978  H -4.65776 -2.02543 -0.59871  H -6.01728 -1.08914 -0.00475 |
| --- | --- |

Form **C** R=NH_2_, E(a.u.) = -645.4147686

| 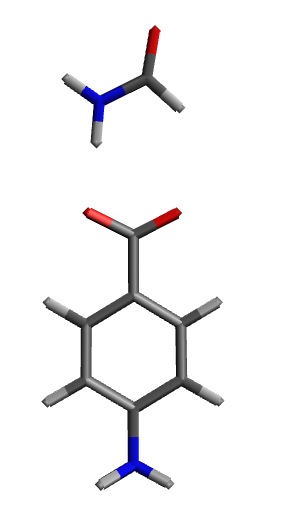 | O 5.83655 -0.52973 0.13362  C 4.62668 -0.33642 0.06521  N 4.02697 0.85447 -0.00488  H 3.89861 -1.16204 0.05392  H 3.00235 0.93280 -0.05121  H 4.59772 1.68169 0.00336  O 1.24050 -1.10829 -0.10361  C 0.65672 -0.00451 -0.08575  O 1.21263 1.12769 -0.09550  C -0.86352 -0.01245 -0.04685  C -1.57834 -1.20902 -0.04433  C -2.96361 -1.22812 -0.00567  C -3.68895 -0.03286 0.03538  C -2.98021 1.17287 0.03153  C -1.59478 1.17353 -0.00860  H -1.02329 -2.13784 -0.07146  H -3.49479 -2.17375 -0.00724  N -5.08306 -0.04216 0.01937  H -3.52414 2.11085 0.05894  H -1.05213 2.10966 -0.00857  H -5.50080 -0.88066 0.38552  H -5.51224 0.77759 0.41365 |
| --- | --- |

Form **C** R=OMe, E(a.u.) = -704.5635032

| 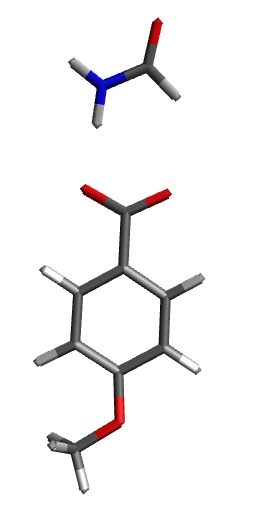 | O -6.30913 0.36947 0.13459  C -5.09258 0.22572 0.06691  N -4.44434 -0.94033 0.00550  H -4.39922 1.08052 0.04898  H -3.41824 -0.97821 -0.04002  H -4.97973 -1.79085 0.02042  O -1.74207 1.11287 -0.11907  C -1.11155 0.03620 -0.08781  O -1.61400 -1.11972 -0.08378  C 0.40909 0.11443 -0.04921  C 1.06689 1.34608 -0.02674  C 2.44674 1.42657 0.00909  C 3.21533 0.26016 0.02279  C 2.57987 -0.97992 0.00011  C 1.18982 -1.03455 -0.03526  H 0.46985 2.24812 -0.03839  H 2.95265 2.38369 0.02639  O 4.56242 0.43392 0.05781  H 3.14885 -1.89852 0.00937  H 0.68904 -1.99325 -0.05312  C 5.39417 -0.70624 0.07321  H 5.25555 -1.31419 -0.82411  H 5.21168 -1.32206 0.95721  H 6.41551 -0.33598 0.09991 |
| --- | --- |

Form **C** R=Me, E(a.u.) = -629.3601162

| 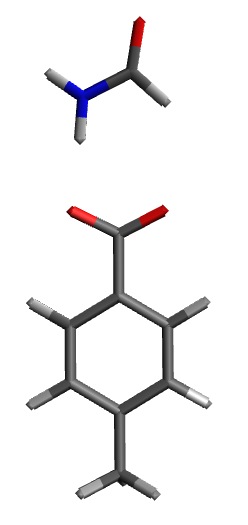 | O -5.85224 -0.53287 -0.12136  C -4.64271 -0.33772 -0.05759  N -4.04489 0.85461 0.00897  H -3.91330 -1.16222 -0.04806  H -3.02113 0.93486 0.05237  H -4.61686 1.68098 0.00200  O -1.25208 -1.10749 0.10572  C -0.67242 -0.00317 0.08245  O -1.22481 1.12892 0.09290  C 0.85317 -0.01121 0.03655  C 1.56260 -1.21029 0.02971  C 2.94989 -1.22107 -0.01223  C 3.67968 -0.03185 -0.04802  C 2.96708 1.16713 -0.04634  C 1.57886 1.17648 -0.00351  H 1.00499 -2.13744 0.05507  H 3.47739 -2.16906 -0.01952  C 5.18097 -0.04553 -0.05991  H 3.50755 2.10706 -0.08055  H 1.03394 2.11114 -0.00352  H 5.56825 -0.88855 -0.63290  H 5.57743 -0.13569 0.95476  H 5.58629 0.87152 -0.48720 |
| --- | --- |

Form **C** R=H, E(a.u.) = -590.0678335

| 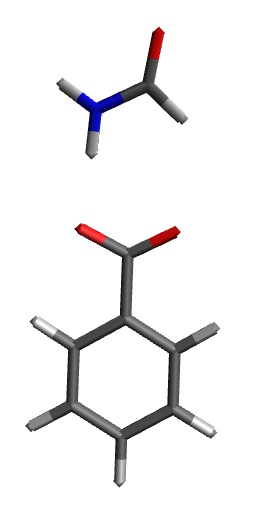 | O -5.35475 -0.52147 -0.13191  C -4.14543 -0.32976 -0.05728  N -3.54485 0.86118 0.01218  H -3.41863 -1.15640 -0.03910  H -2.52166 0.93866 0.06320  H -4.11407 1.68932 -0.00351  O -0.75618 -1.10987 0.12546  C -0.17473 -0.00730 0.09550  O -0.72148 1.12672 0.11038  C 1.35277 -0.02041 0.03262  C 2.05265 -1.22547 0.02035  C 3.44075 -1.24324 -0.03715  C 4.15105 -0.04851 -0.08446  C 3.46377 1.16038 -0.07287  C 2.07559 1.17017 -0.01439  H 1.48736 -2.14727 0.05692  H 3.97033 -2.18849 -0.04527  H 5.23338 -0.05957 -0.12969  H 4.01110 2.09482 -0.10899  H 1.52688 2.10226 -0.00403 |
| --- | --- |

Form **C** R=F, E(a.u.) = -689.3201853

| 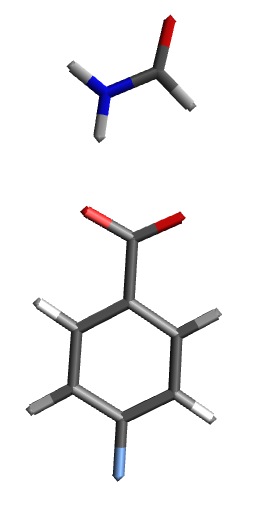 | O 5.81481 -0.53235 0.13262  C 4.60613 -0.33626 0.06180  N 4.00978 0.85717 -0.00411  H 3.87629 -1.16027 0.04454  H 2.98724 0.93839 -0.05321  H 4.58204 1.68321 0.01035  O 1.21260 -1.10842 -0.11538  C 0.63585 -0.00364 -0.09071  O 1.18413 1.12923 -0.10108  C -0.89091 -0.01253 -0.04185  C -1.59419 -1.21518 -0.03561  C -2.98200 -1.23986 0.00737  C -3.65555 -0.03374 0.04497  C -2.99985 1.18265 0.04125  C -1.61178 1.17904 -0.00280  H -1.03313 -2.13927 -0.06551  H -3.53759 -2.16806 0.01175  F -5.00548 -0.04421 0.08679  H -3.56885 2.10221 0.07162  H -1.06370 2.11104 -0.00762 |
| --- | --- |

Form **C** R=Cl, E(a.u.) = -1049.703148

| 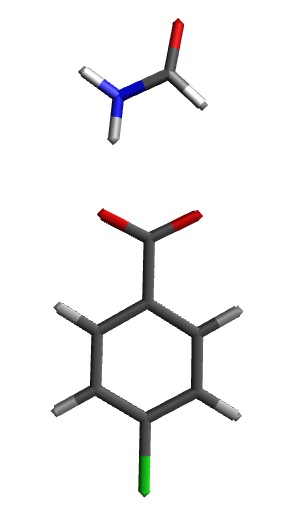 | O -6.27437 -0.53865 0.13951  C -5.06609 -0.34097 0.06871  N -4.47155 0.85341 0.00140  H -4.33516 -1.16406 0.05269  H -3.44952 0.93629 -0.04786  H -5.04499 1.67864 0.01451  O -1.66685 -1.10905 -0.10933  C -1.09448 -0.00245 -0.08970  O -1.64375 1.12927 -0.09831  C 0.43374 -0.00787 -0.05010  C 1.13996 -1.20799 -0.04855  C 2.52756 -1.22922 -0.01289  C 3.21305 -0.02381 0.02277  C 2.54082 1.18947 0.02274  C 1.15300 1.18397 -0.01418  H 0.58255 -2.13456 -0.07588  H 3.06947 -2.16473 -0.01267  Cl 4.96322 -0.03412 0.06919  H 3.09270 2.11872 0.05072  H 0.60546 2.11651 -0.01532 |
| --- | --- |

Form **C** R=CF_3_, E(a.u.) = -927.1295493

| 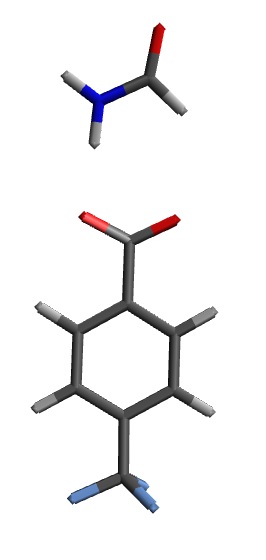 | O 6.99292 -0.56088 0.11998  C 5.78530 -0.35641 0.06247  N 5.19724 0.84159 -0.00069  H 5.04960 -1.17544 0.05670  H 4.17595 0.93095 -0.04101  H 5.77581 1.66330 0.00203  O 2.37300 -1.10488 -0.09105  C 1.81175 0.00648 -0.07535  O 2.36659 1.13423 -0.08238  C 0.28025 0.01149 -0.04378  C -0.43053 -1.18770 -0.04658  C -1.81483 -1.19394 -0.02047  C -2.51139 0.01219 0.01056  C -1.81752 1.21555 0.01507  C -0.42943 1.20745 -0.01214  H 0.12521 -2.11487 -0.06978  H -2.35404 -2.13294 -0.02336  C -4.00533 -0.02241 0.03020  H -2.35529 2.15313 0.03964  H 0.12493 2.13553 -0.00938  F -4.48308 -0.72586 1.07224  F -4.51284 -0.60664 -1.07064  F -4.55881 1.19308 0.10481 |
| --- | --- |

Form **C** R=NO_2_, E(a.u.) = -794.5622712

| 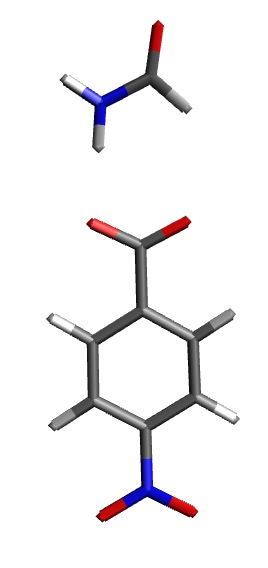 | O 6.57832 -0.53384 0.15598  C 5.37024 -0.33986 0.08228  N 4.77382 0.85273 -0.00657  H 4.64131 -1.16497 0.08182  H 3.75333 0.93379 -0.05813  H 5.34553 1.67923 -0.01046  O 1.95184 -1.11624 -0.10420  C 1.38936 -0.00661 -0.09473  O 1.93453 1.12435 -0.11250  C -0.14420 -0.00843 -0.05617  C -0.84712 -1.21251 -0.05080  C -2.23088 -1.22992 -0.01724  C -2.90920 -0.01876 0.01402  C -2.23947 1.19746 0.01121  C -0.85561 1.19036 -0.02521  H -0.28598 -2.13598 -0.07355  H -2.78401 -2.15691 -0.01404  N -4.37924 -0.02411 0.05149  H -2.79926 2.12015 0.03599  H -0.30121 2.11789 -0.03012  O -4.94996 -1.09979 0.03387  O -4.95605 1.04753 0.09859 |
| --- | --- |

"Double" complexes

Acid R=NO_2_, anion R=NO_2_, E(a.u.) = -1419.739527

| 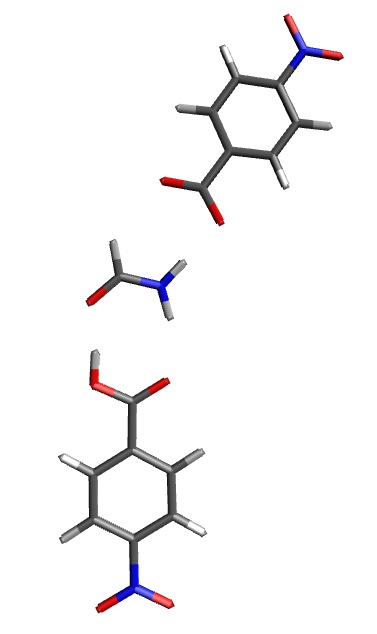 | O -1.00748 2.85515 -0.00000  C 0.05860 2.22024 0.00000  N 0.17364 0.90314 0.00001  H 1.02352 2.74098 0.00000  H 1.10505 0.47053 0.00001  H -0.66716 0.34147 0.00001  O 3.43816 1.87257 0.00001  C 3.62738 0.64238 0.00000  O 2.75619 -0.26188 0.00000  C 5.08393 0.16565 0.00000  C 6.12508 1.09250 0.00000  C 7.44651 0.68040 0.00000  C 7.71549 -0.68177 -0.00000  C 6.70191 -1.63051 -0.00000  C 5.38775 -1.19509 0.00000  H 5.87494 2.14380 0.00000  H 8.26023 1.38984 -0.00000  N 9.11513 -1.13385 -0.00000  H 6.94919 -2.68129 -0.00000  H 4.57335 -1.90551 0.00000  O 9.99136 -0.28815 -0.00001  O 9.33049 -2.33249 -0.00000  H -2.47413 2.18824 -0.00000  O -3.44407 1.91452 -0.00001  C -3.57936 0.60845 0.00000  O -2.65761 -0.18254 0.00001  C -5.00916 0.15801 0.00000  C -6.06287 1.07027 -0.00000  C -7.37316 0.62243 -0.00000  C -7.60564 -0.74375 -0.00000  C -6.57622 -1.67238 0.00000  C -5.27222 -1.21016 0.00000  H -5.85473 2.12957 -0.00001  H -8.20278 1.31226 -0.00001  N -9.00299 -1.22958 -0.00000  H -6.80054 -2.72776 0.00001  H -4.44444 -1.90441 0.00001  O -9.89134 -0.40081 -0.00001  O -9.18458 -2.43083 0.00000 |
| --- | --- |

Acid R=NO_2_, anion R=H, E(a.u.) = -1215.245294

| 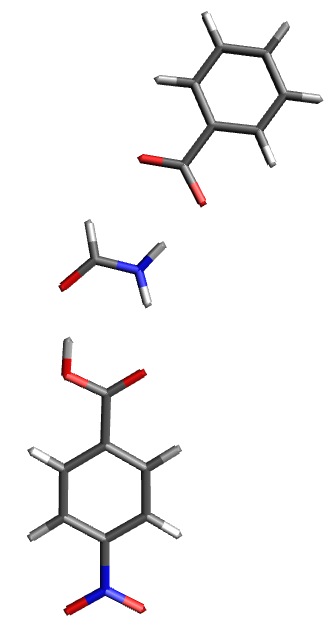 | O 0.35566 2.65715 0.01501  C 1.38800 1.96662 0.02173  N 1.43448 0.64628 0.04780  H 2.37831 2.43609 0.00475  H 2.34422 0.16397 0.05102  H 0.56523 0.13035 0.06348  O 4.70639 1.44289 -0.03574  C 4.85256 0.20493 0.00520  O 3.93264 -0.65329 0.05608  C 6.28135 -0.33536 -0.00746  C 7.36754 0.53673 -0.04345  C 8.67005 0.05344 -0.05638  C 8.90386 -1.31733 -0.03470  C 7.82784 -2.19747 0.00140  C 6.52793 -1.70688 0.01567  H 7.16997 1.60013 -0.06097  H 9.50393 0.74467 -0.08382  H 9.91833 -1.69712 -0.04564  H 8.00331 -3.26649 0.01868  H 5.68244 -2.38102 0.04553  H -1.13788 2.07284 0.01608  O -2.12348 1.85599 0.00776  C -2.33701 0.56101 0.03256  O -1.46558 -0.28435 0.06605  C -3.79176 0.19800 0.01549  C -4.78724 1.17314 -0.01017  C -6.12250 0.80682 -0.02508  C -6.43888 -0.54246 -0.01503  C -5.46852 -1.53252 0.01007  C -4.13859 -1.15130 0.02592  H -4.51339 2.21742 -0.01821  H -6.90844 1.54588 -0.04450  N -7.86335 -0.94061 -0.03172  H -5.75650 -2.57229 0.01702  H -3.35514 -1.89495 0.04607  O -8.69835 -0.05813 -0.04823  O -8.11978 -2.12817 -0.02803 |
| --- | --- |

Acid R=NO_2_, anion R=NMe_2_, E(a.u.) = -1349.144435

| 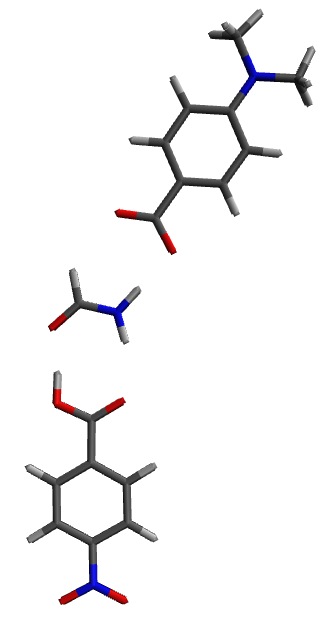 | O -1.12701 2.83984 0.00934  C -0.05640 2.20917 -0.00426  N 0.06652 0.89419 -0.02443  H 0.90626 2.73340 -0.00013  H 1.00477 0.46611 -0.03379  H -0.77143 0.32876 -0.03018  O 3.27420 1.88069 -0.00731  C 3.49950 0.65140 -0.03222  O 2.62542 -0.25872 -0.04887  C 4.94825 0.20057 -0.04345  C 5.99160 1.12217 -0.01890  C 7.32038 0.73093 -0.02261  C 7.67428 -0.63197 -0.06293  C 6.61859 -1.56458 -0.07109  C 5.29728 -1.14744 -0.06671  H 5.74280 2.17501 0.00921  H 8.08418 1.49424 0.00554  N 8.99876 -1.03593 -0.10847  H 6.82554 -2.62466 -0.08157  H 4.50441 -1.88405 -0.07631  C 10.04160 -0.07264 0.16404  C 9.32146 -2.42816 0.10828  H 9.98912 0.33260 1.18282  H 9.99759 0.76303 -0.53491  H 11.00949 -0.55088 0.03717  H 8.82094 -3.06553 -0.62110  H 9.04477 -2.77698 1.11164  H 10.39187 -2.56899 -0.01831  H -2.58118 2.17219 0.01168  O -3.55354 1.89872 0.01696  C -3.69264 0.59385 -0.00530  O -2.77430 -0.20068 -0.02922  C -5.12438 0.14763 0.00155  C -6.17507 1.06302 0.02598  C -7.48685 0.61961 0.03178  C -7.72408 -0.74569 0.01306  C -6.69771 -1.67747 -0.01137  C -5.39221 -1.21949 -0.01711  H -5.96305 2.12146 0.04026  H -8.31410 1.31207 0.05045  N -9.12274 -1.22688 0.01924  H -6.92550 -2.73202 -0.02536  H -4.56669 -1.91621 -0.03606  O -10.00854 -0.39547 0.04023  O -9.30862 -2.42747 0.00289 |
| --- | --- |

Acid R=H, anion R=NO_2_, E(a.u.) = -1215.251132

| 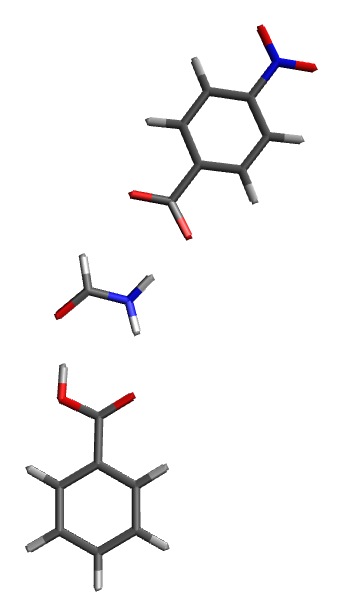 | O -2.34754 2.55443 -0.00000  C -1.25119 1.97591 -0.00000  N -1.06774 0.66537 -0.00000  H -0.31323 2.54464 -0.00001  H -0.11453 0.28537 -0.00000  H -1.87895 0.06091 0.00000  O 2.16233 1.84256 -0.00000  C 2.41661 0.62420 0.00000  O 1.59657 -0.32636 0.00001  C 3.89749 0.22695 0.00000  C 4.88875 1.20704 0.00000  C 6.22991 0.86434 0.00000  C 6.57027 -0.48181 0.00000  C 5.60761 -1.48240 -0.00000  C 4.27261 -1.11578 -0.00000  H 4.58482 2.24407 0.00000  H 7.00522 1.61550 0.00000  N 7.99173 -0.85884 0.00000  H 5.90896 -2.51903 -0.00000  H 3.49551 -1.86654 -0.00000  O 8.82188 0.03226 0.00000  O 8.27091 -2.04429 -0.00000  H -3.79959 1.78775 -0.00000  O -4.74905 1.46841 0.00000  C -4.81295 0.15135 -0.00000  O -3.83604 -0.57407 -0.00000  C -6.20746 -0.37872 0.00000  C -7.31518 0.46844 0.00000  C -8.59746 -0.06183 0.00000  C -8.78081 -1.43971 0.00000  C -7.67936 -2.28857 -0.00000  C -6.39778 -1.75948 -0.00000  H -7.16712 1.53881 0.00000  H -9.45395 0.59976 0.00001  H -9.78176 -1.85222 0.00000  H -7.82064 -3.36152 -0.00000  H -5.53132 -2.40622 -0.00000 |
| --- | --- |

Acid R=H, anion R=H, E(a.u.) = -1010.756677

| 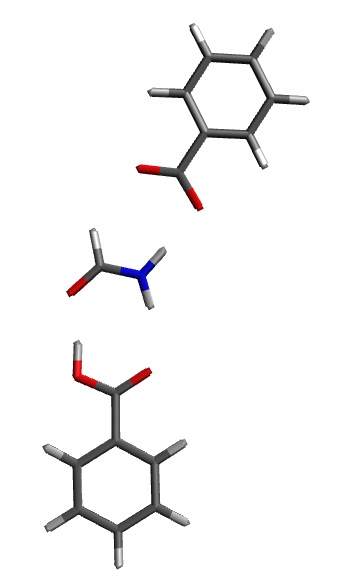 | O -0.96798 -2.42626 -0.00238  C 0.09381 -1.78437 0.00040  N 0.19989 -0.46577 0.00348  H 1.06358 -2.29540 0.00044  H 1.13119 -0.03016 0.00582  H -0.64571 0.08926 0.00380  O 3.41552 -1.46315 0.00231  C 3.64681 -0.23720 0.00258  O 2.79046 0.68493 0.00463  C 5.11200 0.19685 0.00034  C 6.12819 -0.75688 0.00043  C 7.46417 -0.37697 -0.00115  C 7.80301 0.97186 -0.00299  C 6.79767 1.93313 -0.00327  C 5.46328 1.54533 -0.00156  H 5.84663 -1.80112 0.00183  H 8.24199 -1.13103 -0.00094  H 8.84376 1.27223 -0.00425  H 7.05549 2.98548 -0.00481  H 4.67239 2.28311 -0.00169  H -2.45447 -1.75132 -0.00223  O -3.42401 -1.49432 -0.00238  C -3.57852 -0.18494 -0.00033  O -2.65495 0.60711 0.00143  C -5.00775 0.24498 -0.00041  C -6.04906 -0.68273 -0.00147  C -7.36765 -0.25165 -0.00145  C -7.65462 1.10840 -0.00045  C -6.61997 2.03779 0.00057  C -5.30168 1.60764 0.00063  H -5.82059 -1.73861 -0.00229  H -8.17129 -0.97638 -0.00221  H -8.68367 1.44463 -0.00047  H -6.84189 3.09705 0.00132  H -4.48715 2.31880 0.00145 |
| --- | --- |

Acid R=H, anion R=NMe_2_, E(a.u.) = -1144.655781

| 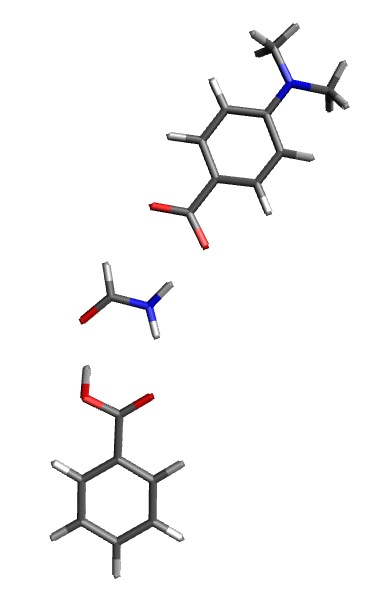 | O -2.46423 -2.54078 0.11652  C -1.36488 -1.97277 0.01888  N -1.17332 -0.66969 -0.09565  H -0.43080 -2.54726 0.02111  H -0.21383 -0.30091 -0.17007  H -1.98002 -0.05974 -0.10401  O 2.01644 -1.87804 -0.15515  C 2.29521 -0.66030 -0.18957  O 1.46531 0.28716 -0.26596  C 3.76186 -0.27187 -0.13405  C 4.76598 -1.23451 -0.07225  C 6.10826 -0.89543 -0.01814  C 6.51691 0.45260 -0.03233  C 5.50008 1.42631 -0.07532  C 4.16472 1.06111 -0.13055  H 4.47614 -2.27734 -0.06130  H 6.83952 -1.68861 0.03537  N 7.85741 0.80411 -0.01972  H 5.74785 2.47761 -0.06640  H 3.40101 1.82708 -0.16513  C 8.84882 -0.20131 0.28997  C 8.22465 2.18048 0.22570  H 8.80159 -1.03085 -0.41599  H 8.73708 -0.60964 1.30275  H 9.83936 0.23877 0.20735  H 7.92014 2.53034 1.22066  H 7.77989 2.84387 -0.51649  H 9.30408 2.28042 0.14480  H -3.90509 -1.77241 0.10800  O -4.85597 -1.45213 0.11520  C -4.92275 -0.13853 0.02878  O -3.94881 0.58640 -0.05034  C -6.31842 0.39014 0.03698  C -7.42404 -0.45571 0.11945  C -8.70699 0.07313 0.12359  C -8.89311 1.44848 0.04615  C -7.79371 2.29612 -0.03567  C -6.51157 1.76830 -0.04042  H -7.27363 -1.52408 0.17970  H -9.56178 -0.58761 0.18735  H -9.89452 1.85993 0.04964  H -7.93700 3.36716 -0.09575  H -5.64662 2.41401 -0.10333 |
| --- | --- |

Acid R=NMe_2_, anion R=NO_2_, E(a.u.) = -1349.154867

| 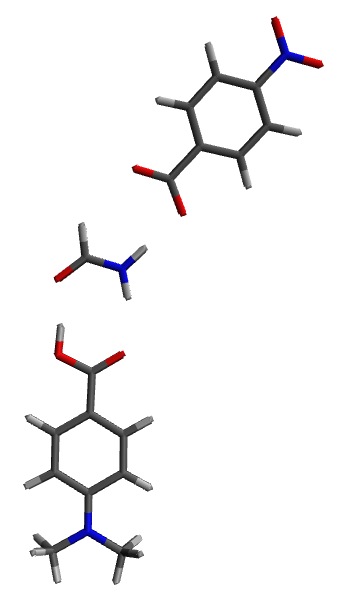 | O -0.90823 3.05501 -0.03337  C 0.15787 2.42425 -0.04232  N 0.27614 1.10616 -0.05839  H 1.12180 2.94909 -0.03670  H 1.20553 0.67264 -0.06399  H -0.56756 0.54610 -0.06276  O 3.63907 1.98610 0.05979  C 3.76553 0.74996 -0.00733  O 2.85197 -0.10748 -0.08240  C 5.19748 0.20010 0.00150  C 6.28471 1.07083 0.05978  C 7.58318 0.59111 0.06744  C 7.78303 -0.78201 0.01684  C 6.72245 -1.67608 -0.04111  C 5.43257 -1.17340 -0.04867  H 6.09017 2.13316 0.09835  H 8.43110 1.25770 0.11152  N 9.15728 -1.30517 0.02417  H 6.91587 -2.73744 -0.07914  H 4.58404 -1.84099 -0.09439  O 10.07545 -0.50687 0.07942  O 9.31197 -2.51223 -0.02562  H -2.41935 2.32654 -0.02565  O -3.37499 2.04441 -0.01604  C -3.47901 0.72263 -0.03089  O -2.51027 -0.01930 -0.05436  C -4.87068 0.23221 -0.01572  C -5.97303 1.08915 0.01336  C -7.26408 0.60177 0.02930  C -7.51720 -0.78832 0.01612  C -6.39669 -1.64948 -0.01316  C -5.11462 -1.14230 -0.02859  H -5.81384 2.15847 0.02430  H -8.08244 1.30522 0.05269  N -8.79275 -1.27996 0.03085  H -6.53112 -2.72041 -0.02365  H -4.27204 -1.82048 -0.05092  C -9.92343 -0.37804 0.06911  C -9.02627 -2.70795 0.02747  H -9.94274 0.28300 -0.80151  H -9.91617 0.24384 0.96865  H -10.84174 -0.95694 0.06945  H -8.58926 -3.19256 0.90496  H -8.61201 -3.18340 -0.86564  H -10.09560 -2.89459 0.03985 |
| --- | --- |

Acid R=NMe_2_, anion R=H, E(a.u.) =-1144.660315

| 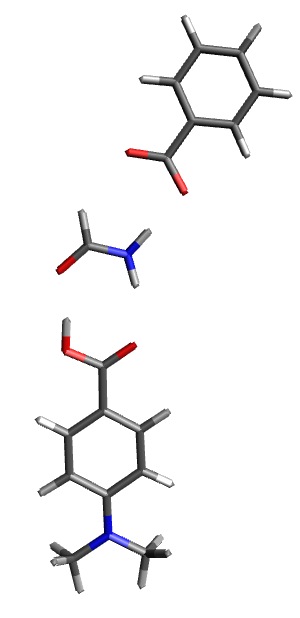 | O 0.50698 -2.74345 -0.03659  C 1.52464 -2.03475 -0.04398  N 1.54388 -0.71201 -0.07649  H 2.52567 -2.48283 -0.02136  H 2.44170 -0.21214 -0.08029  H 0.66093 -0.21721 -0.09553  O 4.85088 -1.43826 0.07213  C 4.97325 -0.19928 -0.00251  O 4.03938 0.63952 -0.09208  C 6.39336 0.36579 0.01604  C 7.49269 -0.48794 0.08501  C 8.78733 0.01594 0.10288  C 9.00017 1.38945 0.05363  C 7.91106 2.25140 -0.01505  C 6.61913 1.74010 -0.03464  H 7.31090 -1.55376 0.12400  H 9.63145 -0.66130 0.15568  H 8.07005 3.32249 -0.05385  H 5.76402 2.40038 -0.09020  H 10.00837 1.78553 0.06848  H -1.04556 -2.13467 -0.03606  O -2.02224 -1.93074 -0.02312  C -2.23673 -0.62303 -0.05365  O -1.33515 0.19823 -0.09710  C -3.66556 -0.25230 -0.02962  C -4.68918 -1.20125 0.01124  C -6.01775 -0.82821 0.03483  C -6.39052 0.53450 0.01861  C -5.34922 1.48939 -0.02197  C -4.02781 1.09577 -0.04584  H -4.43699 -2.25244 0.02494  H -6.77143 -1.60026 0.06689  N -7.70393 0.91443 0.04128  H -5.57648 2.54452 -0.03419  H -3.24742 1.84445 -0.07669  C -8.75202 -0.08114 0.09890  C -8.05918 2.31694 0.03655  H -8.68044 -0.69425 1.00170  H -8.72504 -0.74716 -0.76773  H -9.71686 0.41642 0.10779  H -7.68825 2.82462 -0.85776  H -7.66435 2.83899 0.91272  H -9.14060 2.41113 0.04947 |
| --- | --- |

Acid R=NMe_2_, anion R=NMe_2_, E(a.u.) = -1278.559301

| 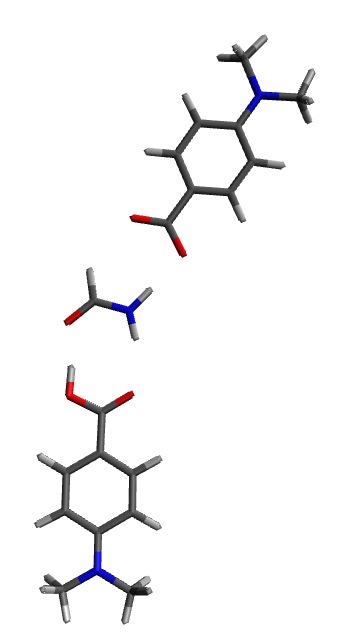 | O 0.50698 -2.74345 -0.03659  C 1.52464 -2.03475 -0.04398  N 1.54388 -0.71201 -0.07649  H 2.52567 -2.48283 -0.02136  H 2.44170 -0.21214 -0.08029  H 0.66093 -0.21721 -0.09553  O 4.85088 -1.43826 0.07213  C 4.97325 -0.19928 -0.00251  O 4.03938 0.63952 -0.09208  C 6.39336 0.36579 0.01604  C 7.49269 -0.48794 0.08501  C 8.78733 0.01594 0.10288  C 9.00017 1.38945 0.05363  C 7.91106 2.25140 -0.01505  C 6.61913 1.74010 -0.03464  H 7.31090 -1.55376 0.12400  H 9.63145 -0.66130 0.15568  H 8.07005 3.32249 -0.05385  H 5.76402 2.40038 -0.09020  H 10.00837 1.78553 0.06848  H -1.04556 -2.13467 -0.03606  O -2.02224 -1.93074 -0.02312  C -2.23673 -0.62303 -0.05365  O -1.33515 0.19823 -0.09710  C -3.66556 -0.25230 -0.02962  C -4.68918 -1.20125 0.01124  C -6.01775 -0.82821 0.03483  C -6.39052 0.53450 0.01861  C -5.34922 1.48939 -0.02197  C -4.02781 1.09577 -0.04584  H -4.43699 -2.25244 0.02494  H -6.77143 -1.60026 0.06689  N -7.70393 0.91443 0.04128  H -5.57648 2.54452 -0.03419  H -3.24742 1.84445 -0.07669  C -8.75202 -0.08114 0.09890  C -8.05918 2.31694 0.03655  H -8.68044 -0.69425 1.00170  H -8.72504 -0.74716 -0.76773  H -9.71686 0.41642 0.10779  H -7.68825 2.82462 -0.85776  H -7.66435 2.83899 0.91272  H -9.14060 2.41113 0.04947 |
| --- | --- |

Values of Laplacian and density at hydrogen bond critical point for **D** complexes

**Table S1.** The ∇^2^*ρ* values at hydrogen bond critical point in **D** complexes

| acid side | | | anion side | | |
| --- | --- | --- | --- | --- | --- |
| R | interaction | ∇^2^*ρ* | ∇^2^*ρ* | interaction | R |
| NO_2_ | OH···O | 0.138 | 0.104 | NH···O | NO_2_ |
|  | NH···O | 0.065 | 0.0259 | CH···O |  |
| NO_2_ | OH···O | 0.139 | 0.107 | NH···O | H |
|  | NH···O | 0.063 | 0.0279 | CH···O |  |
| NO_2_ | OH···O | 0.140 | 0.109 | NH···O | NMe_2_ |
|  | NH···O | 0.064 | 0.0287 | CH···O |  |
| H | OH···O | 0.133 | 0.102 | NH···O | NO_2_ |
|  | NH···O | 0.065 | 0.0254 | CH···O |  |
| H | OH···O | 0.135 | 0.103 | NH···O | H |
|  | NH···O | 0.063 | 0.0299 | CH···O |  |
| H | OH···O | 0.134 | 0.108 | NH···O | NMe_2_ |
|  | NH···O | 0.063 | 0.0270 | CH···O |  |
| NMe_2_ | OH···O | 0.122 | 0.100 | NH···O | NO_2_ |
|  | NH···O | 0.071 | 0.0201 | CH···O |  |
| NMe_2_ | OH···O | 0.127 | 0.103 | NH···O | H |
|  | NH···O | 0.068 | 0.0268 | CH···O |  |
| NMe_2_ | OH···O | 0.126 | 0.102 | NH···O | NMe_2_ |
|  | NH···O | 0.065 | 0.0306 | CH···O |  |

**Table S2.** The *ρ* values at hydrogen bond critical point in **D** complexes

| acid side | | | anion side | | |
| --- | --- | --- | --- | --- | --- |
| R | interaction | *ρ* | *ρ* | interaction | R |
| NO_2_ | OH···O | 0.054 | 0.035 | NH···O | NO_2_ |
|  | NH···O | 0.019 | 0.0087 | CH···O |  |
| NO_2_ | OH···O | 0.055 | 0.037 | NH···O | H |
|  | NH···O | 0.019 | 0.0093 | CH···O |  |
| NO_2_ | OH···O | 0.055 | 0.038 | NH···O | NMe_2_ |
|  | NH···O | 0.019 | 0.0096 | CH···O |  |
| H | OH···O | 0.050 | 0.034 | NH···O | NO_2_ |
|  | NH···O | 0.019 | 0.0086 | CH···O |  |
| H | OH···O | 0.051 | 0.035 | NH···O | H |
|  | NH···O | 0.019 | 0.0099 | CH···O |  |
| H | OH···O | 0.051 | 0.037 | NH···O | NMe_2_ |
|  | NH···O | 0.019 | 0.0091 | CH···O |  |
| NMe_2_ | OH···O | 0.046 | 0.034 | NH···O | NO_2_ |
|  | NH···O | 0.021 | 0.0067 | CH···O |  |
| NMe_2_ | OH···O | 0.047 | 0.035 | NH···O | H |
|  | NH···O | 0.020 | 0.0090 | CH···O |  |
| NMe_2_ | OH···O | 0.048 | 0.035 | NH···O | NMe_2_ |
|  | NH···O | 0.019 | 0.0100 | CH···O |  |

| *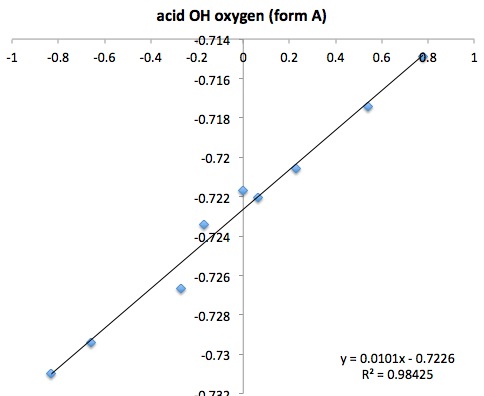*  *a* | *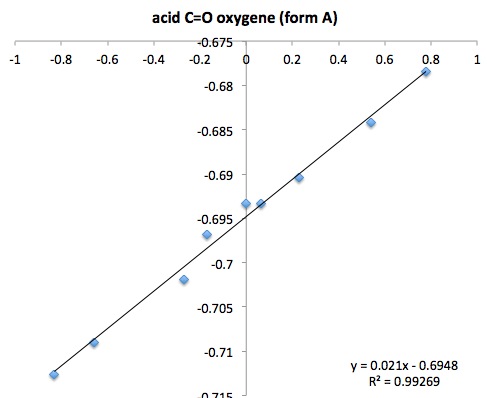*  *b* |
| --- | --- |
| *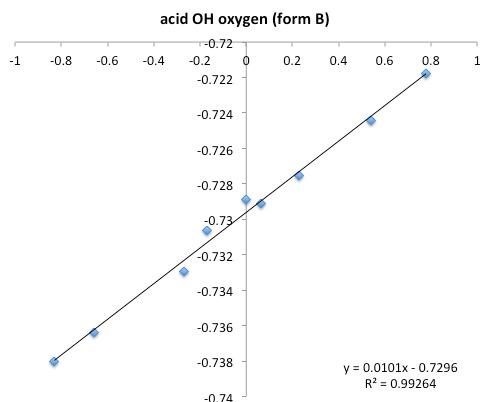*  *c* | *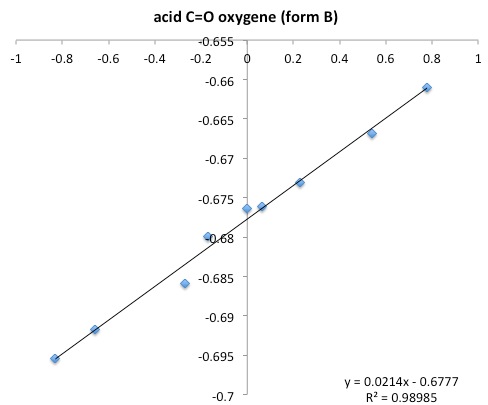*  *d* |
| *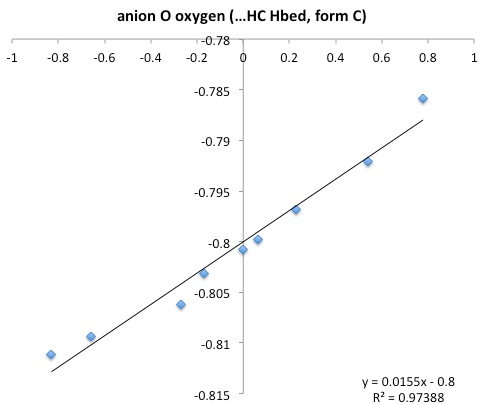*  *e* | *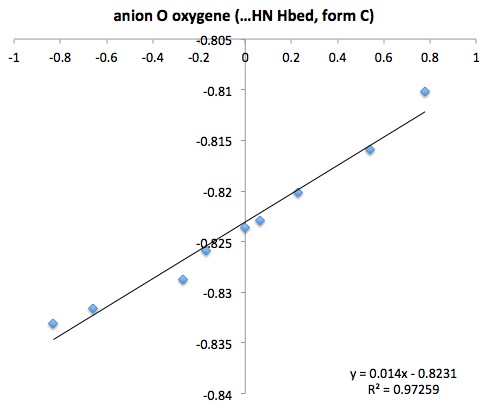*  *f* |

**Chart S1.** The natural charges at oxygen atoms of the benzoic acid/benzoate in associates *a*) OH oxygen in acid in associate A, *b*) CO oxygen in acid in form A, *c*) OH oxygen in acid in form B, *d*) CO oxygen in acid in form B, *e*) O···HC hydrogen bonded oxygen in anion in form C, *f*) O···HN hydrogen bonded oxygen in anion in form C

| *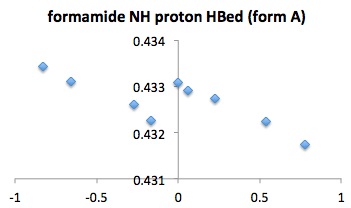*  *a* | *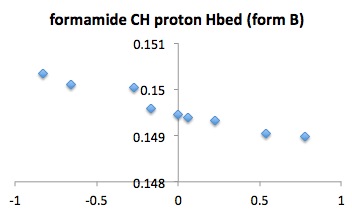*  *b* |
| --- | --- |
| *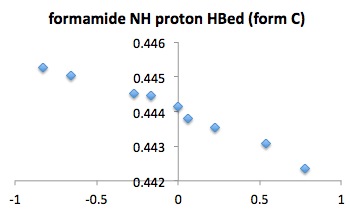*  *c* | *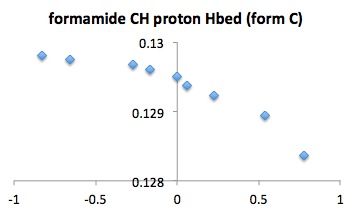*  *d* |

**Chart S2.** The natural charges at hydrogen bonded protons of formamide in associates **A**-**C**, *a*) NH hydrogen bonded proton in **A**, *b*) CH hydrogen bonded proton in **B**, *c*) NH hydrogen bonded proton in **C**, *d*) CH hydrogen bonded proton in **C**

**The differences in electron density at formamide heavy atoms in D complexes *vs* its respective atoms in A or C**

| acid side | anion side | nitrogen  form **D** vs **A** | oxygen  form **D** vs **A** | carbon  form **D** vs **A** |
| --- | --- | --- | --- | --- |
| NO_2_ | NO_2_ | -0.01369 | -0.04058 | -0.01185 |
| NO_2_ | H | -0.01419 | -0.04402 | -0.01301 |
| NO_2_ | NMe_2_ | -0.01461 | -0.04592 | -0.01375 |
| H | NO_2_ | -0.01376 | -0.04028 | -0.01144 |
| H | H | -0.0143 | -0.04368 | -0.01299 |
| H | NMe_2_ | -0.0147 | -0.04549 | -0.01319 |
| NMe_2_ | NO_2_ | -0.01366 | -0.03971 | -0.00981 |
| NMe_2_ | H | -0.01467 | -0.04407 | -0.01218 |
| NMe_2_ | NMe_2_ | -0.01528 | -0.04563 | -0.0135 |

| acid side | anion side | NH proton  (acid side)  form **D** vs **A** | CH proton  (anion side)  **D** vs **C** | NH proton  (anion side)  form **D** vs **C** |
| --- | --- | --- | --- | --- |
| NO_2_ | NO_2_ | -0.01665 | 0.023 | 0.00295 |
| NO_2_ | H | -0.01815 | 0.02408 | 0.00297 |
| NO_2_ | NMe_2_ | -0.01884 | 0.02444 | 0.00282 |
| H | NO_2_ | -0.01674 | 0.02047 | 0.00212 |
| H | H | -0.01827 | 0.02316 | 0.00169 |
| H | NMe_2_ | -0.01885 | 0.02107 | 0.00207 |
| NMe_2_ | NO_2_ | -0.01520 | 0.0136 | 0.00133 |
| NMe_2_ | H | -0.01694 | 0.01859 | 0.00088 |
| NMe_2_ | NMe_2_ | -0.01794 | 0.02167 | 0.00055 |
